# PD-1 blockade restores helper activity of tumor-infiltrating, exhausted PD-1^hi^CD39^+^ CD4 T cells

**DOI:** 10.1172/jci.insight.142513

**Published:** 2021-01-25

**Authors:** Camille-Charlotte Balança, Anna Salvioni, Clara-Maria Scarlata, Marie Michelas, Carlos Martinez-Gomez, Carlos Gomez-Roca, Victor Sarradin, Marie Tosolini, Carine Valle, Frédéric Pont, Gwénaël Ferron, Laurence Gladieff, Sébastien Vergez, Agnès Dupret-Bories, Eliane Mery, Philippe Rochaix, Jean-Jacques Fournié, Jean-Pierre Delord, Christel Devaud, Alejandra Martinez, Maha Ayyoub

**Affiliations:** 1 Cancer Research Center of Toulouse, INSERM UMR 1037, Toulouse, France.; 2 Immune Monitoring Core Facility,; 3 Department of Surgery, and; 4 Department of Medical Oncology, Institut Claudius Regaud, Institut Universitaire du Cancer de Toulouse, Toulouse, France.; 5 Technological Pole and Bioinformatic Platform, Cancer Research Center of Toulouse, INSERM UMR 1037, Toulouse, France.; 6 Department of Surgery, Centre Hospitalier Universitaire, Institut Universitaire du Cancer de Toulouse, Toulouse, France.; 7 Université Toulouse III Paul Sabatier, Toulouse, France.; 8 Department of Pathology, Institut Claudius Regaud, Institut Universitaire du Cancer de Toulouse, Toulouse, France.

**Keywords:** Immunology, Cancer immunotherapy, T cells

## Abstract

Tumor antigen–specific CD4 T cells accumulate at tumor sites, evoking their involvement in antitumor effector functions in situ. Contrary to CD8 cytotoxic T lymphocyte exhaustion, that of CD4 T cells remains poorly appreciated. Here, using phenotypic, transcriptomic, and functional approaches, we characterized CD4 T cell exhaustion in patients with head and neck, cervical, and ovarian cancer. We identified a CD4 tumor-infiltrating lymphocyte (TIL) population, defined by high PD-1 and CD39 expression, which contained high proportions of cytokine-producing cells, although the quantity of cytokines produced by these cells was low, evoking an exhausted state. Terminal exhaustion of CD4 TILs was instated regardless of TIM-3 expression, suggesting divergence with CD8 T cell exhaustion. scRNA-Seq and further phenotypic analyses uncovered similarities with the CD8 T cell exhaustion program. In particular, PD-1^hi^CD39^+^ CD4 TILs expressed the exhaustion transcription factor TOX and the chemokine CXCL13 and were tumor antigen specific. In vitro, PD-1 blockade enhanced CD4 TIL activation, as evidenced by increased CD154 expression and cytokine secretion, leading to improved dendritic cell maturation and consequently higher tumor-specific CD8 T cell proliferation. Our data identify exhausted CD4 TILs as players in responsiveness to immune checkpoint blockade.

## Graphical abstract

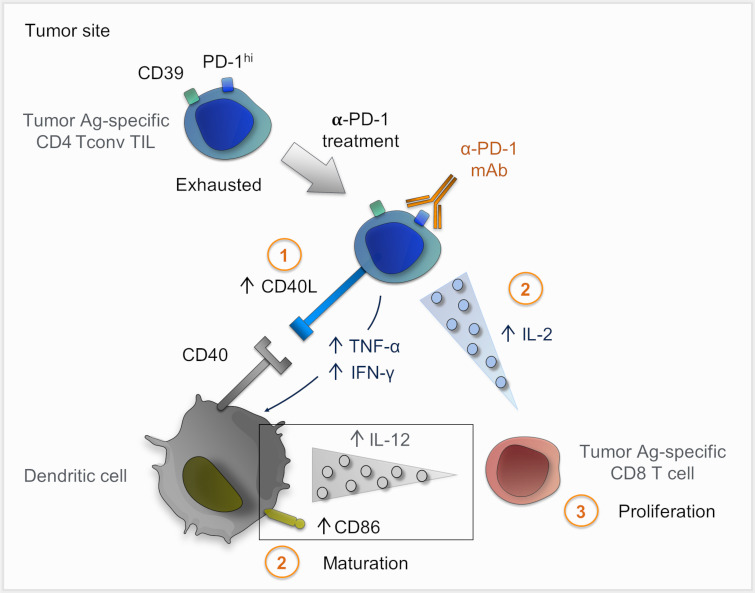

## Introduction

By virtue of their direct antitumor cytotoxic effector functions, CD8 T cells have been at the core of antitumor immunity investigations. We, and others, have nonetheless shown that circulating CD4 T cells specific for tumor antigens (Ags) are detectable in patients bearing Ag^+^ tumors (1, 2). The contribution of CD4 T cells to CD8 T cell priming, whereby CD4 T cells help foster clonal expansion and acquisition of CD8 T cell memory and effector functions, is generally acknowledged (3). In addition to their foreseeable role at the priming phase and despite the lack of MHC class II molecule expression in the vast majority of tumor cells, CD4 T cells are thought to exert effector functions through for instance IFN-γ secretion (4). We showed that tumor Ag–specific CD4 T cells accumulate at tumor sites, where they are found at higher proportions than in the circulation, supporting their involvement in direct or indirect antitumor effector functions in situ (1, 5). Recently, the presence of tumor-specific CD4 T cells, specifically at the tumor site, was shown to be necessary for immune-mediated tumor rejection (6).

The clinical success of immune checkpoint blockade (ICB) roused interest in understanding T cell exhaustion as a means of developing more potent immunotherapy combinations and identifying biomarkers of response. The study of T cell exhaustion in patients with cancer, mainly focused on CD8 T cells, uncovered exhaustion as a marker of tumor specificity and of response to ICB (7–9). Here, we undertook the analysis of tumor-infiltrating CD4 T cell exhaustion in patients with cancer and present data positioning exhausted CD4 T cells as players of response to ICB.

## Results

### Coexpression of high PD-1 and CD39 defined a population of functionally exhausted CD4 TILs.

We showed that circulating tumor Ag–specific CD8 T cells in patients with epithelial malignancies express intermediate levels of PD-1 and TIGIT (7). When they infiltrate tumors, they sequentially express CTLA-4 and then TIM-3 and concomitantly upregulate PD-1 and TIGIT, leading to an exhausted population that we named quadruple positive (QP), i.e., expressing the 4 immune checkpoints (ICs) (7). To investigate CD4 T cell exhaustion, because virtually all CD4 tumor-infiltrating lymphocytes (TILs) do not express CD45RA, we assessed IC expression in memory conventional FOXP3^-^ CD4 T cells (CD4 Tconvs; Supplemental Figure 1A; supplemental material available online with this article; https://doi.org/10.1172/jci.insight.142513DS1) from TILs of patients with head and neck cancer, cervical cancer, and ovarian cancer (OC). The proportion of cells expressing PD-1 was the highest, followed by that of cells expressing TIGIT, CTLA-4, and then TIM-3 (Supplemental Figure 1B). Comparison of IC expression among tumor types showed that PD-1^+^ cells were detected at higher proportions in OC samples than in head and neck cancer samples (Supplemental Figure 1B). Expression of TIGIT, CTLA-4, and TIM-3 was systematically associated with that of PD-1 (Supplemental Figure 1B), and correlation analyses revealed significant positive correlations between proportions of cells expressing PD-1 and those expressing each of the other ICs (Supplemental Figure 1B). IC coexpression analysis showed that the proportion of QP cells was much lower among CD4 TILs (range, 0.23%–43.81%; median, 3.28%; Supplemental Figure 2A) than CD8 TILs (range, 0.7%–62%; median, 20.69%; ref. 7). In CD8 TILs, we showed that IC accumulation is an indicator of chronic stimulation, whereby the QP population, identified by TIM-3 expression, represents terminally exhausted tumor Ag–specific T cells (7, 10). The reduced proportion of QP CD4 TILs could be compatible with lower terminal exhaustion or could imply that terminally exhausted CD4 TILs express different markers. Nonetheless, as in CD8 TILs, sequential CTLA-4 and then TIM-3 expression in CD4 Tconv TILs was concomitant to enhanced expression of PD-1 and TIGIT (Supplemental Figure 2B). Exhausted CD8 TILs express the ectonucleotidase CD39 (8) and its expression is virtually limited to QP CD8 TILs (7). In CD4 TILs, CD39 was likewise expressed in a significant proportion of QP cells (Supplemental Figure 2C). Nonetheless, among TIM-3^–^ CD4 TIL subpopulations, significant proportions of cells expressed CD39 (Supplemental Figure 2C).

**Figure 1:**
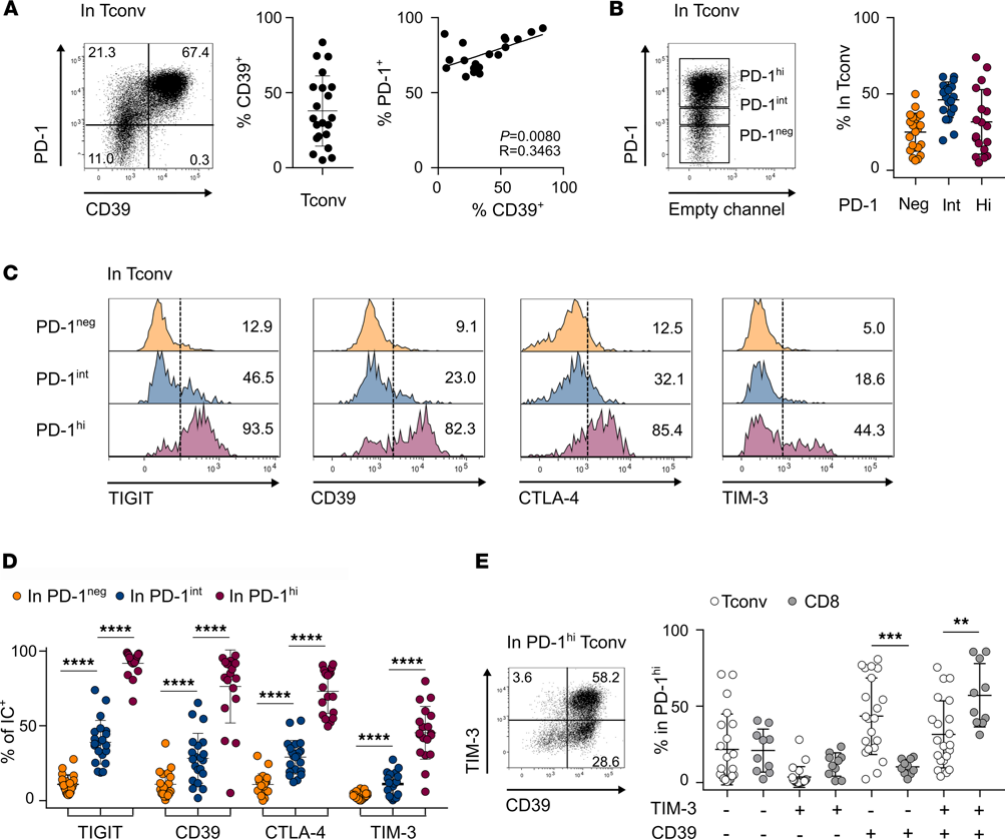
CD39 expression in CD4^+^ Tconv TILs. Isolated CD4^+^ and CD8^+^ TILs were stained ex vivo and analyzed by flow cytometry. (**A**) Dot plot shows PD-1 versus CD39 expression in cells gated on memory CD4 Tconvs as shown in Supplemental Figure 1A. Proportions of CD39^+^ cells (center, *n* = 21) and correlation between the proportion of PD-1^+^ and CD39^+^ (right, *n* = 19) in CD4 Tconvs. (**B**) Dot plot shows PD-1 expression and gates defining PD-1^neg^, PD-1^int^, and PD-1^hi^ cells within CD4 Tconvs. Proportions of PD-1^neg^, PD-1^int^, and PD-1^hi^ cells in CD4 Tconvs (*n* = 21). (**C** and **D**) Histogram plots in **C** show TIGIT, CD39, CTLA-4, and TIM-3 expression in PD-1^neg^, PD-1^int^, and PD-1^hi^ CD4 Tconvs and proportions are summarized in **D** (*n* = 21). (**E**) Dot plot shows TIM-3 versus CD39 expression in PD-1^hi^ CD4 Tconvs. Proportions of TIM-3^–^CD39^–^, TIM-3^+^CD39^–^, TIM-3^–^CD39^+^, and TIM-3^+^CD39^+^ cells among PD-1^hi^ CD4 Tconvs (*n* = 21) and PD-1^hi^ CD8^+^ TILs (*n* = 10). Data are presented as mean ± SD. **P < 0.01; ***P < 0.001; ****P < 0.0001. Pearson’s correlation (**A**), 2-tailed paired *t* test or Wilcoxon (**D**), and 2-tailed unpaired *t* test (**E**) were used to compare variables. Tconvs, conventional FOXP3^-^ CD4 T cells; TILs, tumor-infiltrating lymphocytes.

We therefore investigated in more depth CD39 expression in CD4 TILs. On average, 37.9% of CD4 Tconv TILs expressed CD39; all CD39^+^ cells expressed high levels of PD-1 (PD-1^hi^); and CD39 expression was positively correlated to that of PD-1 (Figure 1A). No difference was found in the proportion of CD39^+^ cells among tumor types (Supplemental Figure 2D). Because high PD-1 expression is associated with exhaustion, we analyzed expression of ICs, including CD39, among PD-1^neg^, PD-1^int^, and PD-1^hi^ CD4 Tconv TILs (Figure 1B). The PD-1^hi^ population contained the highest proportions of cells expressing the investigated ICs (Figure 1, C and D). Most PD-1^hi^ cells expressed TIGIT, CTLA-4, and CD39, whereas the proportion of TIM-3^+^ cells was lower (Figure 1, C and D). We compared, in CD8 and CD4 Tconv TILs, CD39 and TIM-3 expression among PD-1^hi^ cells. Within the PD-1^hi^ CD4 Tconv fraction, CD39 expression occurred alone or combined with that of TIM-3, whereas cells expressing TIM-3 only were rarely detected (Figure 1E). In contrast, within CD8 PD-1^hi^ cells, CD39 and TIM-3 were systematically coexpressed (Figure 1E). These data show divergence in CD39 and TIM-3 expression between CD4 and CD8 TILs and suggest that exhausted CD4 TILs could be identified by expression of CD39 rather than TIM-3.

**Figure 2:**
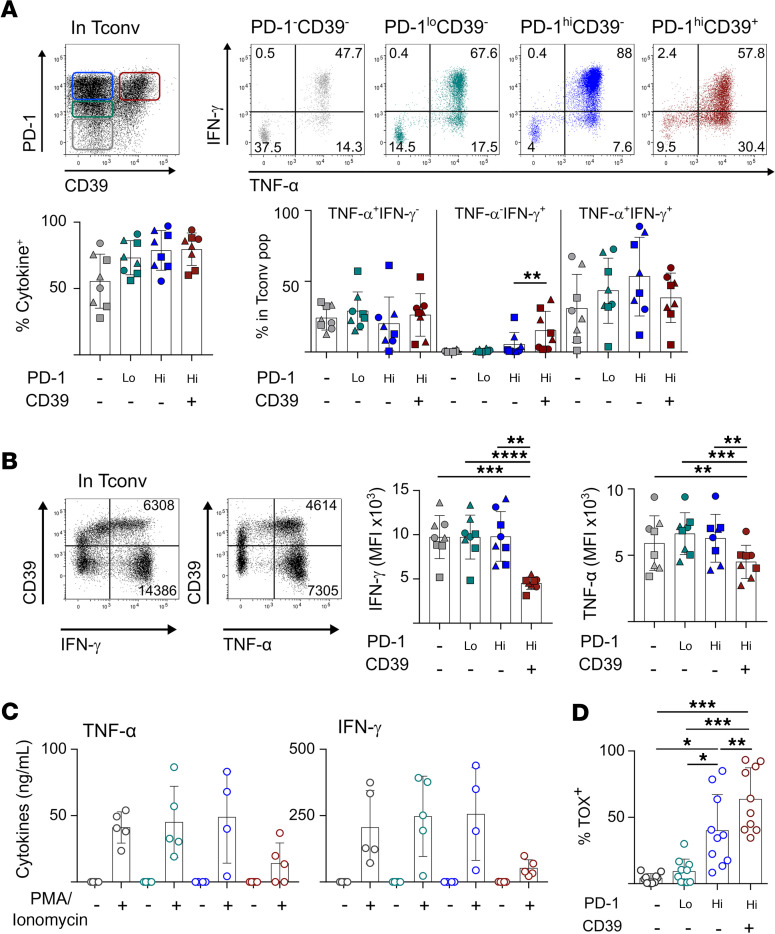
PD-1^hi^CD39^+^ tumor-infiltrating CD4 Tconvs are functionally exhausted. (**A** and **B**) Isolated CD4^+^ TILs were stimulated in vitro with PMA/ionomycin and stained and analyzed by flow cytometry. (**A**) Top left dot plot shows PD-1 versus CD39 expression in CD4 Tconvs. IFN-γ versus TNF-α expression is shown in the indicated CD4 Tconv populations. Proportions of cytokine^+^ (IFN-γ and/or TNF-α; bottom left) and TNF-α^+^IFN-γ^–^, TNF-α^–^IFN-γ^+^, and TNF-α^+^IFN-γ^+^ cells in PD-1^–^CD39^–^, PD-1^lo^CD39^–^, PD-1^hi^CD39^–^, and PD-1^hi^CD39^+^ subsets and according to TIM-3 expression (*n* = 8). (**B**) Dot plots show CD39 versus IFN-γ or TNF-α in CD4 Tconvs. Numbers in dot plots correspond to MFI of cytokine staining. MFI of IFN-γ and TNF-α staining in IFN-γ^+^ and TNF-α^+^ cells, respectively, are summarized for Tconv subpopulations defined as in **A** (*n* = 8). Samples in **A** and **B** are shown according to the proportion of TIM-3^+^ cells among PD-1^hi^CD39^+^ CD4 Tconv (<50% TIM-3^+^, square; ≥50% TIM-3^+^, triangle; unknown, round) (**C**) CD4 Tconv TIL subsets PD-1^–^CD39^–^ (gray), PD-1^lo^CD39^–^ (green), PD-1^hi^CD39^–^ (blue), and PD-1^hi^CD39^+^ (red) were sorted, expanded in vitro for 10 days, and restimulated or not with PMA/ionomycin overnight. TNF-α and IFN-γ secretion was quantified by CBA in the supernatant (*n* = 5). (**D**) Isolated CD4^+^ TILs were stained ex vivo and analyzed by flow cytometry. Proportions of TOX^+^ cells in each Tconv subset are summarized (*n* = 10). Data are presented as mean ± SD. **P < 0.01; ***P < 0.001; ****P < 0.0001. *P* values were determined using the Wilcoxon (**A** and **B**) and 2-tailed paired *t* tests (**D**). Bonferroni’s correction was applied to account for multiple testing in **D** and significance level was adjusted accordingly (*P < 0.0083; **P < 0.00166; ***P < 0.000166). Tconvs, conventional FOXP3^-^ CD4 T cells; CBA, cytometric bead array; TILs, tumor-infiltrating lymphocytes.

To explore the functional consequences of CD39 expression in CD4 Tconv TILs, we stimulated CD4 TILs with PMA and ionomycin and assessed IFN-γ and TNF-α production in Tconv TIL subsets defined according to PD-1 and CD39 expression, i.e., PD-1^–^CD39^–^, PD-1^lo^CD39^–^, PD-1^hi^CD39^–^, and PD-1^hi^CD39^+^ (Figure 2A). The 2 CD4 Tconv PD-1^hi^ populations contained the highest proportions of cytokine^+^ cells, implying advanced differentiation. Assessment of the differentiation stage of CD4 Tconv TILs according to PD-1 expression confirmed this assumption. The PD-1^–^ population contained the highest proportion of central memory (CD45RA^–^CCR7^+^) cells, whereas the PD-1^hi^ population was enriched in effector memory (CD45RA^–^CCR7^–^) cells (Supplemental Figure 3). Within PD-1^hi^ populations, CD39^–^ cells contained high proportions of polyfunctional IFN-γ^+^TNF-α^+^ cells. Their proportions were lower in CD39^+^ cells, in favor of IFN-γ single-positive cells, indicative of terminal exhaustion (Figure 2A) (11). Analysis of IFN-γ^+^ and TNF^+^ populations showed that their respective MFIs were lower in PD-1^hi^CD39^+^ cells than in all other populations (Figure 2B), suggesting that these cells produce lower amounts of cytokines. The proportion of cytokine^+^ cells and their MFIs did not vary according to TIM-3 expression in PD-1^hi^CD39^+^ cells (Figure 2, A and B). Ex vivo cell sorting of the 4 CD4 Tconv TIL populations, defined according to PD-1 and CD39 expression, followed by PMA/ionomycin stimulation and secreted cytokine quantification, showed that PD-1^hi^CD39^+^ cells produced lower amounts of IFN-γ and TNF-α as well as IL-2 (Supplemental Figure 4), features that corroborated their functional exhaustion. In vitro–expanded PD-1^hi^CD39^+^ cultures produced lower amounts of cytokines after restimulation compared with the other subpopulations (Figure 2C). Collectively, these results put forward CD39, rather than TIM-3, as a marker of terminal exhaustion in CD4 Tconv TILs. In agreement with this assumption, expression of the CD8 T cell exhaustion transcription factor TOX (12, 13) was highest in PD-1^hi^CD39^+^ CD4 Tconv TILs (Supplemental Figure 5 and Figure 2D).

### scRNA-Seq revealed exhaustion, tumor residency, and late differentiation signatures in CD39^+^ CD4 TILs.

**Figure 3:**
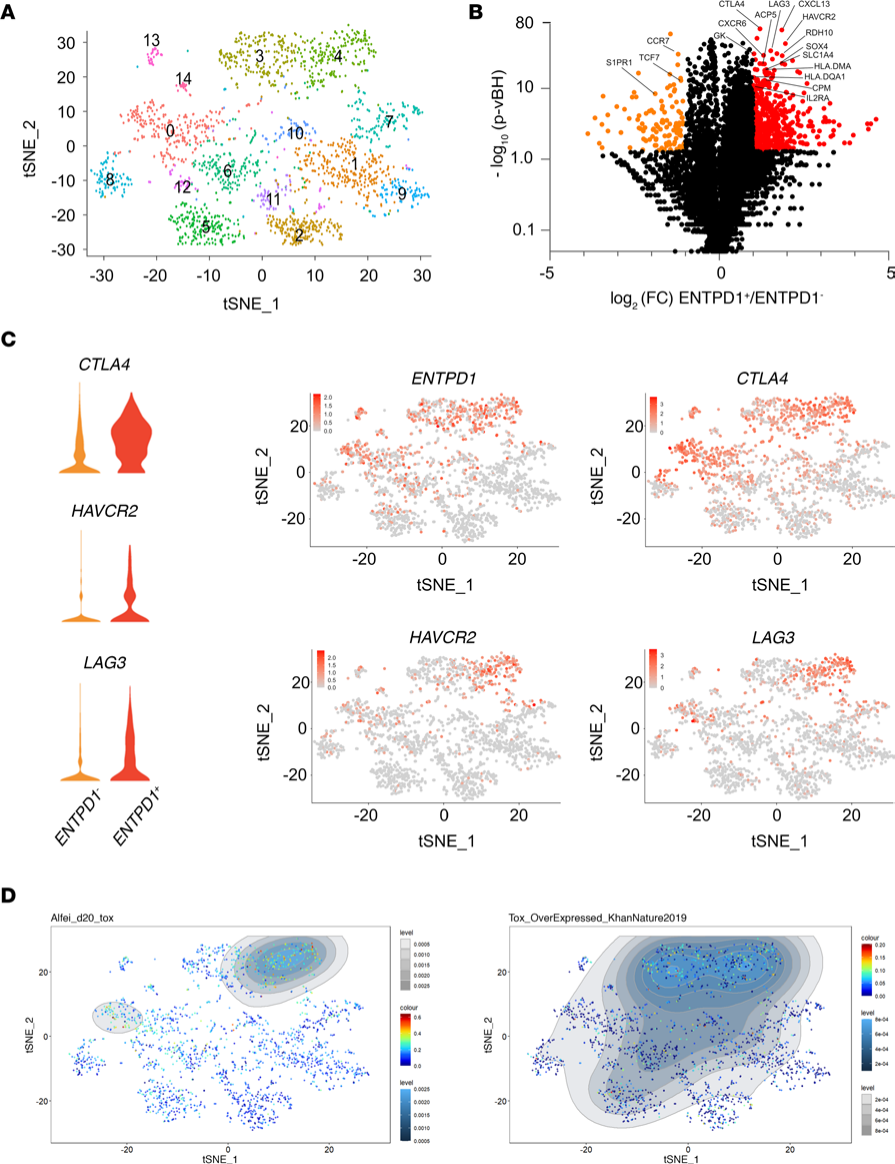
scRNA-Seq of tumor-infiltrating CD4 Tconvs. CD45^+^ cells isolated ex vivo from 4 head and neck cancer specimens were subjected to scRNA-Seq. (**A**) t-SNE plot of 2060 CD4 Tconvs color-coded by their associated cluster. (**B**) Results of differential expression analysis of scRNA-Seq data from *ENTPD1*^+^ versus *ENTPD1*^–^ CD4 Tconvs are shown in a volcano plot. Genes significantly upregulated in *ENTPD1*^+^ cells are shown in red and those significantly downregulated are shown in orange. (**C**) Violin plots showing the expression of *CTLA4*, *HAVCR2,* and *LAG3* in *ENTPD1*^–^ and *ENTPD1*^+^ cells (left) and t-SNE plots color-coded by levels of expression (gray to red) of *ENTPD1, CTLA4, HAVCR2*, and *LAG3* (right). (**D**) t-SNE plots showing analysis of TOX exhaustion gene sets (left,ref. 15, and right, ref. 12) using Single-Cell Signature Explorer Viewer. Tconvs, conventional FOXP3^-^ CD4 T cells; TILs, tumor-infiltrating lymphocytes.

To further characterize PD-1^hi^CD39^+^ CD4 Tconv TILs, we performed scRNA-Seq of total CD45^+^ cells isolated ex vivo from 4 head and neck tumors. Of 9872 CD45^+^ cells, 2060 (20.8%) were identified as CD4 Tconvs based on the coexpression of *CD3D*, *CD3E*, and *CD3G*; the detection of barcoded anti-CD4 mAbs; and absence of *FOXP3*. Clustering of CD4 T cells identified 15 clusters (Figure 3A). Differential mRNA expression analysis between *ENTPD1* (CD39) positive and negative CD4 Tconv (Figure 3B) identified 291 differentially expressed genes. Among ICs, *CTLA4*, *HAVCR2*, and *LAG3* were upregulated in *ENTPD1*^+^ cells (Figure 3C). *CTLA4* was expressed in the same clusters as *ENTPD1*, whereas *HAVCR2* (TIM-3) was expressed only in a subpopulation of *ENTPD1*^+^ cells (Figure 3C), confirming flow cytometry data. Expression of *LAG3* was detected in the same clusters as *HAVCR2* (Figure 3C). We computed a pseudotime maturation trajectory. Visualizing gene expression levels in cells along the trajectory showed early acquisition of *CTLA4* in comparison with *ENTPD1*, whereas those of *HAVCR2* and *LAG3* appeared at later stages (Supplemental Figure 6). Differential analysis showed that *ENTPD1*^+^ cells expressed activation markers (*IL2RA* and genes encoding proteins of the MHC class II presentation pathway; Supplemental Figure 7A) and genes encoding proteins involved in metabolic pathways (Supplemental Figure 7B), suggesting that CD39^+^ CD4 TILs are activated. Similar to exhausted CD8 TILs (7), *ENTPD1*^+^ CD4 TILs showed loss of early T cell differentiation markers *CCR7* and *TCF7* (TCF-1) (Supplemental Figure 7C), compatible with their advanced differentiation. Among tissue-resident memory (Trm) cell markers expressed by exhausted CD8 TILs (7), CD4 TILs did not express the adhesion molecules CD103 and CD49a (Supplemental Figure 8, A and B) but expressed CD69 (Supplemental Figure 8C). Differential mRNA analysis revealed that *ENTPD1*^+^ cells exhibited *S1PR1* downmodulation (Supplemental Figure 7D). In addition, *ENTPD1*^+^ cells expressed *CXCR6* (Supplemental Figure 7E), which binds CXCL16 produced in secreted and membrane-bound forms by myeloid cells. Among genes expressed in exhausted CD8 TILs (7, 9), *ENTPD1*^+^ cells expressed *SOX4* and *CXCL13* (Supplemental Figure 7F). Finally, in agreement with TOX expression in PD-1^hi^CD39^+^ CD4 TILs (Figure 2D), *ENTPD1*^+^ clusters overexpressed TOX transcriptomic signatures (14), identified by Alfei et al. (15) and Khan et al. (12), after transduction of T cells with TOX-encoding retroviruses (Figure 3D). Altogether, these results show that exhausted CD4 TILs, identified by high PD-1 and CD39 expression, share characteristics of the CD8 T cell exhaustion program. Of note, in agreement with previous reports showing no overlap between CD39 or ICs and CD73 expression in human CD4 T cells (16), we detected no expression of *NT5E* (CD73) in Tconv TILs (Supplemental Figure 7G).

**Figure 4:**
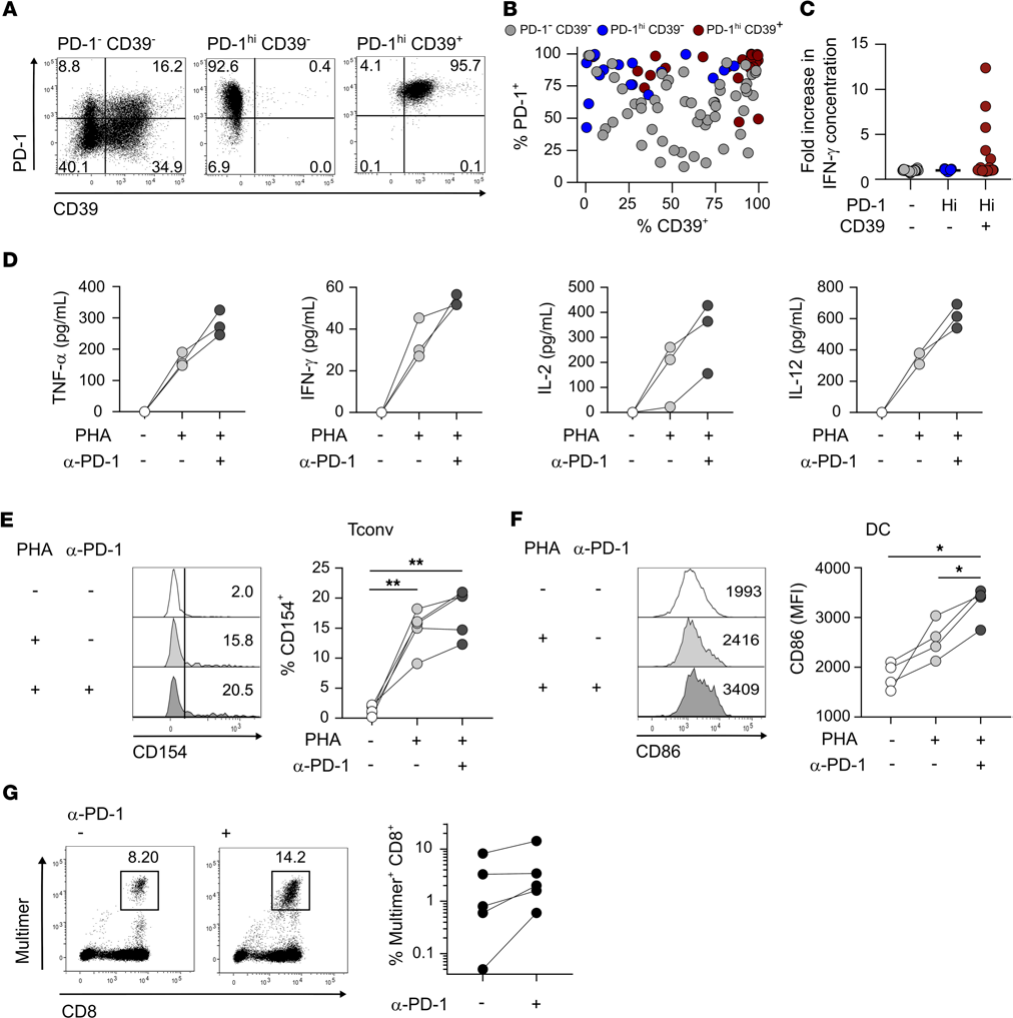
PD-1^hi^CD39^+^ CD4 Tconv TILs encompass tumor Ag–specific cells and respond to PD-1 blockade by enhancing DC-mediated CD8 T cell proliferation. (**A**–**C**) Ex vivo isolated CD4^+^ TILs from one OC NY-ESO-1–seropositive patient were FACS-sorted into PD-1^–^CD39^–^, PD-1^hi^CD39^–^, and PD-1^hi^CD39^+^ CD4 Tconv (CD3^+^CD4^+^CD25^-^CD127^+^) subsets and cloned. (**A** and **B**) Clonal populations were stained and analyzed by flow cytometry. (**A**) PD-1 versus CD39 expression in clones representative of the 3 sorted Tconv populations. (**B**) Proportions of PD-1^+^ and CD39^+^ cells are summarized for all clones derived from PD-1^–^CD39^–^ (*n* = 54), PD-1^hi^CD39^–^ (*n* = 17), and PD-1^hi^CD39^+^ (*n* = 22) subsets. (**C**) IFN-γ concentration in the supernatant was quantified by ELISA for each clone stimulated with NY-ESO-1 peptide pool (fold increase over unstimulated condition) (*n* as in **B**). (**D**–**F**) Ex vivo CD4^+^ TILs (± anti–PD-1 mAbs pretreatment) were cocultured with iDCs in the presence or absence of PHA. (**D**) TNF-α, IFN-γ, IL-2, and IL-12 concentrations were quantified by CBA in the 24-hour supernatants (*n* = 3). (**E**) Histogram plots show CD154 expression in CD4 Tconvs after 6-hours stimulation. Proportions of CD154^+^ cells are summarized (*n* = 5). (**F**) Histogram plots show CD86 expression in DCs in day 2 cultures. MFI of CD86 staining are summarized (*n* = 4). (**G**) CD4^+^ TILs from OC NY-ESO-1–seropositive patients (± anti–PD-1 mAbs pretreatment); autologous iDCs and circulating CD8 T cells were cocultured in the presence of NY-ESO-1 peptides, stained with MHC-I/NY-ESO-1 peptide multimers on day 10, and analyzed by flow cytometry. Examples of dot plots show multimer staining and CD8 expression and proportions of multimer^+^CD8^+^ cells are summarized (*n* = 5). A 2-tailed paired *t* test was used to compare variables (**E** and **F**). Bonferroni’s correction was applied to account for multiple testing (**E** and **F**) and significance level was adjusted accordingly (*P < 0.016; **P < 0.0032). (**E** and **F**). Tconvs, conventional FOXP3^-^ CD4 T cells; TILs, tumor-infiltrating lymphocytes; OC, ovarian cancer; iDCs, immature DCs; CBA, cytometric bead array.

*PD-1^hi^CD39^+^ CD4 Tconv TILs encompassed tumor Ag–specific cells and provided help to CD8 T cells upon PD-1 blockade*. We investigated Ag specificity of tumor-infiltrating CD4 Tconvs according to PD-1 and CD39 expression. We used the cancer testis Ag NY-ESO-1 as a model (5) and selected a patient with OC who exhibited antibody and CD8 T cell responses to the Ag (7). We sorted ex vivo CD4 Tconv TILs into PD-1^–^CD39^–^, PD-1^hi^CD39^–^, and PD-1^hi^CD39^+^ subsets (Figure 2A) and cloned them. In vitro–expanded clonal populations from the PD-1^hi^CD39^–^ and PD-1^hi^CD39^+^ subsets conserved, for the largest part, their ex vivo phenotype (Figure 4, A and B). A proportion of clones derived from sorted PD-1^–^CD39^–^ cells gained some level of PD-1 and/or CD39 expression (Figure 4, A and B). We stimulated all clones with a pool of overlapping peptides encompassing full-length NY-ESO-1. Five of twenty-two clones originating from the PD-1^hi^CD39^+^ subpopulation specifically secreted IFN-γ in response to Ag stimulation, whereas none of the 54 and 17 clones obtained from the PD-1^–^CD39^–^ and PD-1^hi^CD39^–^ subsets, respectively, showed Ag recognition (Figure 4C). To characterize the fine specificity of NY-ESO-1–specific clones, we stimulated them with the 17 single peptides from the NY-ESO-1 peptide pool. We found that the 5 clones represented 3 different fine specificities (Supplemental Figure 9, A–D) and 3 different MHC restrictions as shown by inhibition of the response after stimulation in the presence of blocking mAbs specific for HLA-DR, HLA-DP, and HLA-DQ (Supplemental Figure 9E). These data demonstrate the stability of the exhausted PD-1^hi^CD39^+^ CD4 Tconv phenotype and suggest that this subset encompasses tumor Ag–specific CD4 T cells. Ex vivo stimulation of 3 different CD4 TIL samples, from 2 NY-ESO-1 responder patients, with the NY-ESO-1 peptide pool showed that specific CD154 upregulation and IFN-γ production were nearly exclusively detected in the PD-1^hi^CD39^+^ Tconv subset (Supplemental Figure 10), confirming the PD-1^hi^CD39^+^ phenotype of tumor Ag–specific CD4 Tconv TILs. Of note, in agreement with their exhausted phenotype, only a minority of specific T cells, identified by CD154 upregulation in response to Ag stimulation, produced IFN-γ (Supplemental Figure 10). We suggest that, similar to CD8 TILs (7–9), CD4 T cell exhaustion at the tumor site is instated in tumor Ag–specific T cells as a result of chronic exposure to the Ag.

We investigated whether blocking anti–PD-1 mAbs could directly affect exhausted CD4 T cell helper activity. We first analyzed the impact of PD-1 blockade on the capacity of CD4 Tconv TILs to help DC maturation. Cytokine secretion by CD4 T cells and DCs increased in CD4/immature DCs (iDCs) cocultures when CD4 TILs were pretreated with anti–PD-1 in comparison with cocultures with nontreated CD4 TILs (Figure 4D). In agreement with these results, we found that PD-1 blockade enhanced CD154 (CD40L) expression in CD4 TILs after stimulation (Figure 4E), implying enhanced ability to induce iDC maturation through CD40-CD40L interaction. Accordingly, anti–PD-1 pretreatment of CD4 TILs led to enhanced DC maturation, as evidenced by increased expression of the costimulatory ligand CD86 (Figure 4F) in addition to enhanced IL-12 secretion (Figure 4D). These results show that CD4 TILs can be targeted by anti–PD-1 mAbs, leading to CD4 TIL activation-mediated DC maturation.

We next asked whether anti–PD-1-mediated CD4 TIL rescue, leading to DC maturation, could affect CD8 T cell proliferation. We selected patients with OC with CD8 T cell responses to NY-ESO-1 identified by HLA/peptide multimer staining (7). We preincubated or not CD4 TILs from these patients with anti–PD-1 mAbs and cocultured them with autologous iDCs and circulating CD8 T cells in the presence of NY-ESO-1 peptides (Supplemental Figure 11). Proliferation of NY-ESO-1–specific CD8 T cells in response to the Ag increased in the presence of anti–PD-1 pretreated CD4 TILs in comparison with nontreated CD4 TILs (Figure 4G). We previously found that PD-1^+^ circulating tumor–specific CD8 T cells proliferate after Ag stimulation and PD-1 inhibition (7). Data presented here show that PD-1 blockade could in addition indirectly contribute to tumor-specific CD8 T cell proliferation through in situ reactivation of tumor Ag–specific PD-1^hi^CD39^+^ CD4 Tconv TILs and DC maturation.

## Discussion

Our results set forth exhausted CD4 T cells at the tumor site as players of response to PD-1 blockade. We identified high expression of PD-1 associated with CD39 expression as markers of CD4 T cell exhaustion in situ in 3 epithelial malignancies. PD-1^hi^CD39^+^ CD4 TILs shared characteristics of CD8 T cell exhaustion, in particular, expression of the exhaustion transcription factor TOX (7, 13) and its target genes (12, 15) as well as specificity for tumor Ags (7–9). We showed that although the PD-1^hi^CD39^+^ CD4 TIL population contained high proportions of cytokine-producing cells, the amount of cytokines produced by these cells was lower than that produced by cytokine^+^ cells within the other CD4 TIL subpopulations, supporting their exhausted state. PD-1 blockade in CD4 TILs increased the amount of cytokines produced as well as CD154 upregulation after stimulation, leading to enhanced DC maturation. In a tumor Ag–specific model, we showed that PD-1 blockade in specific PD-1^hi^CD39^+^ CD4 TILs supported proliferation of autologous CD8 T cells specific for the same Ag, implying that CD4 TILs exert helper functions in situ, which could be enhanced by PD-1 blockade. Based on the results presented here and our previous work (7), we propose a model whereby circulating PD-1^+^ tumor–specific CD8 T cells reach the tumor site where they are stimulated by DCs, proliferate, acquire Trm markers to be retained locally, and become terminally exhausted. Under anti–PD-1/PD-L1, CD8 proliferation could be enhanced through direct blockade of PD-1 on CD8 T cells and through reinvigoration of the helper activity of tumor Ag–specific CD4 T cells.

CD8 T cell exhaustion is instated as a means of adapting the immune response to chronic infection and cancer (17, 18). It has been proposed that degrees of exhaustion are linked to disease severity (18). In line with this concept, we showed that ICs are sequentially acquired by tumor–specific CD8 T cells at the tumor site, leading to a terminally exhausted population characterized by TIM-3 expression, the latter being acquired at late stages after PD-1, TIGIT, and CTLA-4 expression acquisition (7). Other studies also found that TIM-3^+^ CD8 TILs are terminally exhausted and encompass tumor-specific cells (8–10). This exhaustion stage was attained by Ag-specific CD8 TILs but not by bystander T cells specific for, for instance, viral Ags (7), suggesting that chronic stimulation in the tumor milieu could be responsible for its instatement. Contrary to CD8 TILs, where CD39 and TIM-3 are virtually always coexpressed, the exhausted PD-1^hi^CD39^+^ CD4 TIL population harbored both TIM-3^–^ and TIM-3^+^ cells. This could reflect a lesser degree of chronic stimulation at the tumor site, compatible with the fact that CD4 TILs recognize Ag-presenting cells (APCs) but not tumor cells that are, for most tumor types, devoid of MHC class II molecules, in contrast to CD8 TILs that are stimulated by both APC and tumors (4). Expression of activation markers and proteins involved in metabolic pathways together with the absence of early memory markers were nonetheless in agreement with the chronic stimulation of exhausted CD4 TILs at the tumor site. Accordingly, PD-1^hi^CD39^+^ CD4 TILs exhibited several features found in exhausted CD8 T cells, including expression of TOX and its target genes (12, 13, 15) as well as that of SOX-4 and CXCL13. SOX-4–mediated CXCL13 production by CD4 T cells is involved in the formation of ectopic lymphoid-like structures (TLSs) in rheumatoid arthritis (19). CXCL13 expression by exhausted CD8 TILs is associated with B cell recruitment and TLS formation (20). Our results imply that exhausted CD4 TILs could also contribute to B cell recruitment and TLS formation within the tumor microenvironment. We and others found that exhausted CD8 TILs express the Trm markers CD103, CD69, and CD49a (7, 8, 21). Of those, exhausted CD4 TILs expressed CD69 only but expressed CXCR6 and exhibited S1PR1 downmodulation. CXCR6 expression could imply enhanced interaction of CD4 TILs with APC, which produce the secreted and membrane-bound forms of the CXCR6 ligand CXCL16, and could be indicative, with CD69 expression and S1PR1 downregulation, of their tissue residency (7, 22, 23). Expression of activation markers, indicative of in situ stimulation and proliferation, and tissue residency are compatible with the accumulation of tumor Ag–specific CD4 T cells at the tumor site where they are found at higher frequencies than in the periphery (5) and could imply that, like exhausted CD8 TILs, exhausted CD4 TILs encompass Ag-specific T cells. Our data show that T cells specific for the cancer testis Ag NY-ESO-1 are solely found within the PD-1^hi^CD39^+^ CD4 TIL population. In a distinct tumor Ag model, CD39^+^ CD4 and CD8 TILs were found to be specific for HPV Ags in HPV-induced malignancies (24).

The presence of exhausted CD8 T cells within tumors, being indicative of an ongoing antitumor immune response, is correlated to clinical responsiveness to ICB (7, 10, 25). Clinical responses are correlated to both exhausted tumor Ag–specific CD8 T cell proliferation (7, 26, 27) in the periphery and at the tumor site and in situ reversal of exhaustion (7). Accumulation of tumor Ag–specific CD4 T cells at tumor sites indicates that they play effector functions through, for instance, IFN-γ secretion (4). Alspach and colleagues showed that the presence of tumor Ag–specific cells, specifically among CD4 TILs, was necessary for tumor regression after immunotherapy by PD-1/PD-L1 axis blockade in a mouse model (6), suggesting that PD-1 blockade directly targets PD-1^+^ CD4 TILs. Our results show that PD-1 blockade, specifically on CD4 TILs, increased their helper functions as evidenced by enhanced DC maturation, leading to increased CD86 expression and IL-12 secretion. In the NY-ESO-1 model, we showed that PD-1 blockade on CD4 TILs led to enhanced proliferation of autologous NY-ESO-1–specific CD8 T cells supporting a major role for CD4 help in CD8 T cell proliferation in situ. This is in agreement with the requirement for a DC and CD8 T cell crosstalk and IL-12 and IFN-γ secretion for responsiveness to immunotherapy (28). Results obtained in the NY-ESO-1 model, whereby specific CD4 T cells are PD-1^hi^CD39^+^, show that anti–PD-1 can restore helper functions of this terminally exhausted population.

The ectonucleotidase activity of CD39 cleaves extracellular ATP and ADP into AMP that is in turn converted into adenosine by the ectonucleotidase CD73. Because ATP is able to enhance DC maturation through purinergic/pyrimidinergic receptors (29), its consumption by CD39 on exhausted CD4 T cells could contribute to, in addition to their exhaustion, their reduced helper activity towards DC. It would be of interest to assess this hypothesis using blocking anti-CD39 mAbs. However, because CD39 is also expressed by DCs, it would be complex in a coculture system, like the one we used here, to assess the specific contribution of CD4 T cells to modulation of ATP concentrations. Adenosine has an immunosuppressive activity and can have direct effects on T cells, leading to reduced proliferation (29). We showed that CD39^+^ CD4 TILs did not express CD73, which was in agreement with previous reports showing no coexpression of CD39 and CD73 in CD4 TILs (16). Therefore, CD39 could not be directly involved in CD4 TIL exhaustion in a cell-intrinsic manner. However, CD39 expression by exhausted CD4 TILs could contribute to adenosine production in the context of the tumor microenvironment, where CD73 is expressed both by immune and tumor cells (29).

## Methods

### Patient and healthy donor samples.

Peripheral blood and tumor samples were collected from 63 patients with head and neck, ovarian, and cervical cancer at the time of surgery for primary disease or for recurrence. The patients included in this study were as follows: 14 patients with stage FIGO IB1-IIIB HPV 16 and/or 18 positive cervical cancer with squamous and adenocarcinoma histology (primary disease, 13 patients; recurrence, 1 patient); 25 patients with stage FIGO IIIB-IVA OC (primary disease, 22 patients; recurrence, 3 patients; high-grade serous carcinoma, 22 patients; clear cell carcinoma, 1 patient; carcinosarcoma, 2 patients); and 24 patients with newly diagnosed (22 patients) or locoregional recurrent (2 patients) head and neck cancer. All patients had histologically documented tumors, were 18 years old or older at the time of study entry, were followed within a standard of care procedure, and had an ECOG performance status of 0–2. Exclusion criteria were as follows: known history of positive test for hepatitis B, hepatitis C, human immunodeficiency, or hantaviruses; any condition contraindicated with blood sampling procedures; pregnancy or breastfeeding; and active suspected or prior documented autoimmune disease or use of immunosuppressive medication. Patients did not receive any therapy during the 3 months prior to study entry. Blood samples from healthy donors (HDs) were obtained from the Etablissement Français du Sang (Toulouse, France).

PBMCs from patients or HDs were isolated by density gradient sedimentation using Ficoll-Hypaque (Sigma-Aldrich). Tumor samples were rapidly transported to the research facility on ice. On arrival, samples were rinsed with PBS (Sigma-Aldrich), subsequently minced on ice to smaller pieces, between 2 mm and 4 mm, dissociated using C-tubes (Miltenyi Biotec) and gentleMACS Octo Dissociator (Program MultiC01_01; Miltenyi Biotec) in Iscove’s Modified Dulbecco’s Medium (IMDM; Sigma-Aldrich), and filtered using a 40 μm nylon mesh (BD Biosciences). PBMCs and tumor single-cell suspensions were cryopreserved in FBS (Gibco) containing 10% DMSO (Sigma-Aldrich).

For scRNA-Seq experiments, 4 head and neck tumors were minced as detailed above, transferred into digestion medium (Tumor Dissociation Kit, human, Miltenyi Biotec), and dissociated using C-tubes and gentleMACS Octo Dissociator (Program h_TDK3, 37°C). Samples were filtered using a 40 μm nylon mesh. Cell suspensions were then centrifuged at 300*g* and 4°C for 7 minutes. The supernatant was discarded and cell pellets were resuspended in 1 mL Red Blood Cell Lysis Buffer (Miltenyi Biotec), incubated for 10 minutes at 4°C, centrifuged, and pellets were resuspended in PBS containing 0.04% BSA (EUROMEDEX). CD45^+^ cells were enriched by positive magnetic selection from single-cell suspensions (CD45 MicroBeads, human, OctoMACS Separator, MS Columns, Miltenyi Biotec). Finally, cells were counted to determine the proportion of live cells. Only samples containing greater than 90% live cells were used for scRNA-Seq experiments.

### Cell purification and phenotypic assessment.

CD4^+^ and CD8^+^ cells were enriched from tumor single-cell suspensions by magnetic positive selection (CD4 or CD8 MicroBeads, human, Miltenyi Biotec) using OctoMACS Separator and MS Columns. Cells were assessed phenotypically by staining with fluorochrome-labeled mAbs specific for CD3, CD4, CD8, CD45RA, PD-1, TIGIT, TIM-3, CD103, CD49a, CD69, and CD39, as indicated. For intracellular and intranuclear staining, cells were fixed, permeabilized, and stained with mAbs specific for CTLA-4, FOXP3, and TOX using the Transcription Factor Staining Buffer Set (eBioscience). mAbs used for flow cytometry analysis are listed in Supplemental Table 1. Cells were analyzed using a BD LSRFortessa X20 flow cytometer and data were analyzed using DIVA software (BD Biosciences) or Flowlogic software (Miltenyi Biotec).

### T cell functional assessment.

CD4^+^ T cells isolated from tumor single-cell suspensions by magnetic positive selection were stimulated with PMA (100 ng/mL; Sigma-Aldrich) and ionomycin (1 g/mL; Sigma-Aldrich) for 6 hours in IMDM supplemented with 1% Penicillin–Streptomycin Solution (Sigma-Aldrich), 1% MEM Non-Essential Amino Acids Solution (Invitrogen), L-glutamine (2 mM, Invitrogen), and 10% human serum (Institut de Biotechnologies Jacques Boy). Brefeldin-A (BFA; 10 μg/mL; Sigma-Aldrich) was added 1 hour after the beginning of the incubation. Cells were then stained, as described above, with mAbs specific for CD3, CD4, CD39, PD-1, and FOXP3, and cytokine production was assessed by intracellular staining using IFN-γ–specific and TNF-α–specific mAbs (Supplemental Table 1). Cells were analyzed by flow cytometry (BD LSRFortessa X20).

For detection of cytokine secretion, isolated CD4^+^ TILs were stained with mAbs specific for CD3, CD4, CD8, PD-1, CD39, CD127, and CD25, and CD4 Tconvs (CD3^+^CD8^–^CD4^+^CD25^–^CD127^+^) were sorted into PD-1^–^CD39^–^, PD-1^lo^CD39^–^, PD-1^hi^CD39^–^, and PD-1^hi^CD39^+^ subsets by flow cytometry cell sorting (BD FACSAria Fusion). Sorted cells were stimulated with PMA/ionomycin overnight. mAbs used for cell sorting are listed in Supplemental Table 2. TNF-α, IFN-γ, and IL-2 were quantified in the supernatant by cytometric bead array (CBA; BD Biosciences).

For cytokine detection after cell expansion, the PD-1^–^CD39^–^, PD-1^lo^CD39^–^, PD-1^hi^CD39^–^, and PD-1^hi^CD39^+^ subsets were sorted as described above and stimulated in vitro with irradiated (35 Gy) PBMCs, phytohemagglutinin (PHA; 1 μg/mL; Sigma-Aldrich) and recombinant human (rh) IL-2 (150 IU/mL; Miltenyi Biotec). Day 20 cultures were stimulated or not with PMA/ionomycin overnight and TNF-α and IFN-γ were quantified in the supernatant by ELISA (Invitrogen).

To characterize ex vivo NY-ESO-1–specific CD4 Tconv TILs, magnetically sorted CD4^+^ TILs were cocultured with magnetically sorted autologous circulating CD14^+^ cells (CD14 MicroBeads, human, Miltenyi Biotec) in the presence or absence of a pool of 17 overlapping 20 to 24 amino acid–long peptides encompassing full-length NY-ESO-1 (1 μM of each peptide in the pool; Peptide 2.0 Inc.; Supplemental Table 3) for 6 hours. BFA (3 μg/mL; eBioscience) was added 1 hour after the beginning of stimulation. After surface staining with mAbs specific for CD3, CD4, PD-1, CD39, and FOXP3, intracellular staining was performed using mAbs specific for IFN-γ and CD154 and cells were analyzed by flow cytometry.

### Droplet-based scRNA-Seq and single-cell gene expression analysis.

CD45^+^ cells (200,000–400,000 cells), prepared as detailed above, were stained with barcoded TotalSeq-A mAbs (Biolegend). Single-cell libraries (3′ gene expression and antibody-derived tag fractions) were generated using the Chromium Controller Instrument and Chromium Single Cell 3′ Library & Gel Bead Kit v3 according to the manufacturer’s protocol (10× Genomics), with some modifications as previously described (30). To detect barcoded TotalSeq-A antibodies, an ADT library was constructed as previously described for CITE-Seq (30). Single-cell library size and quality were confirmed on the Fragment Analyzer (Agilent). KAPA Quantification Kit for Illumina platforms (KAPA Biosystems, Roche) was used to quantify libraries. Samples were pooled in equimolar fashion with desired proportions for the 2 library types (cDNA library fraction at 90% and ADT library at 10%). The libraries were sequenced on a NextSeq 550 (Illumina) in pair-end sequencing 28 bp (read1) × 91 bp (read2) and a single index 8 bp in length. Raw data (FastQ files) for expression and antibody detection were computed with CellRanger 3.0 and the GRCh38 transcriptome as reference (https://support.10xgenomics.com/single-cell-gene-expression/software/pipelines/latest/using/count). Data were then loaded in an R session with the Seurat 3.0 toolkit package involving the normalization and variance stabilization package sctransform (31). Samples were individually filtered using unique molecular identifier (<30,000) and percentage of mitochondrial genes (<0.25%) criteria. Using Seurat, data sets were reduced by PCA, using the first 11 principal components to reduce dimensionality by t-distributed stochastic neighbor embedding (t-SNE). A resolution parameter set the granularity at 1.2 for the clustering by the K-nearest neighbor graph-based clustering approach of Seurat’s FindClusters function. Tconvs were selected with Single-Cell Virtual Cytometer software (32) using the sum of *CD3D*, *CD3E*, and *CD3G* gene expression, CD4-barcoded TotalSeq-A mAbs signal, and absence of *FOXP3* gene expression. Wilcoxon’s *P* values for differentially expressed genes were adjusted with Benjamini-Hochberg at p-vBH greater than 3 and illustrated in a volcano plot. The maturation trajectories of the selected T cells were computed with the Dynverse package (33) using Slingshot tool (34). Trajectories were represented as dendrograms. Expression of genes from the TOX signatures published by Alfei et al. (15) and Khan et al. (12) were analyzed using Single-Cell Signature Explorer Viewer (14).

scRNA-Seq data have been deposited in NCBI’s Gene Expression Omnibus and are accessible through GEO series accession number GSE148162.

### CD4 Tconv TILs cell sorting and cloning and phenotypic and functional characterization of clonal populations.

CD4^+^ cells were magnetically sorted ex vivo from tumor single-cell suspensions, as described above, from 1 patient with OC, who we previously identified as a responder to NY-ESO-1 with detectable CD8 T cell and antibody responses (7). Isolated CD4^+^ T cells were stained with CD3, CD4, CD8, PD-1, CD39, CD127, and CD25 mAbs, and CD4 Tconvs (CD3^+^CD8^–^CD4^+^CD25^–^CD127^+^) were FACS-sorted into PD-1^–^CD39^–^, PD-1^hi^CD39^–^, and PD-1^hi^CD39^+^ subsets (BD FACSAria Fusion). mAbs are listed in Supplemental Table 2. Sorted cells were cloned by limiting dilution in presence of allogeneic irradiated (35 Gy) PBMCs, PHA (1 μg/mL), and rhIL-2 (150 IU/mL). Clones were subsequently expanded by periodic stimulation (every 2 to 3 weeks) under similar conditions. Phenotyping of clones obtained from each subset was performed on day 12 after restimulation cultures using mAbs specific for CD3, CD4, PD-1, and CD39 and flow cytometry analysis.

To identify NY-ESO-1–specific clones, all clones from the PD1^–^CD39^–^, PD1^hi^CD39^–^, and PD1^hi^CD39^+^ subsets were incubated overnight in IMDM supplemented with 10% HS in the presence or absence of the NY-ESO-1 long peptide pool (1 μM) and IFN-γ concentration was assessed in the culture supernatant by ELISA.

For functional assessment of clones by intracellular cytokine and CD154 staining, monocyte-derived iDCs from HLA-matched HDs were used in some experiments. To identify HLA-matched HDs able to present NY-ESO-1 peptides to specific clones, total PBMCs from HDs were incubated overnight at 37°C in the presence or absence of the NY-ESO-1 long peptide pool (1 μM). After thorough washing, clones were added to PBMCs in IMDM 10% HS medium containing BFA (3 μg/mL). After a 3-hour incubation, cells were stained with mAbs specific for CD3, CD4, IFN-γ, and TNF-α and analyzed by flow cytometry. To derive iDCs, CD14^+^ cells were enriched from PBMCs by magnetic positive selection and cultured for 5 days in IMDM 10% HS supplemented with rhIL-4 and GM-CSF, both at 1000 IU/mL (Miltenyi Biotec).

To determine the fine specificity of NY-ESO-1–specific clones, they were stimulated independently with each of the NY-ESO-1 long peptides (1 μM) for 4 hours at 37°C in the presence of HLA-matched iDCs from HDs. BFA (3 μg/mL) was added 1 hour after the beginning of the incubation. After surface staining with mAbs specific for CD3 and CD4, intracellular staining was performed using mAbs specific for IFN-γ, TNF-α, and CD154 and cells were analyzed by flow cytometry.

For inhibition experiments with blocking anti-HLA class II mAbs, iDCs from HLA-matched HDs were preincubated for 1 hour at 37°C with anti-HLA-DR, anti-HLA-DP, or anti-HLA-DQ mAbs (10 μg/mL; Supplemental Table 4), then clones and NY-ESO-1 peptides were added and cultures were supplemented with BFA (3 μg/mL) 1 hour after beginning of stimulation. After 4 hours incubation, IFN-γ and TNF-α production was assessed by intracellular staining and flow cytometry analysis as described above.

### Functional characterization of CD4 TILs after PD-1 blockade.

iDCs were obtained from HD as described above. Isolated CD4^+^ TILs were preincubated or not with anti–PD-1 mAbs (10 μg/mL; Bristol-Myers Squibb) for 30 minutes at 4°C and washed twice with IMDM supplemented with 10% FBS. iDCs (75,000 to 100,000 cells, per well) and anti–PD-1 pretreated or not CD4^+^ TILs (50,000 to 100,000 cells, per well) were cocultured in the presence or absence of PHA (1 μg/mL) in IMDM 10% HS. For the assessment of CD40L expression in CD4 cells, BFA (3 μg/mL) was added 1 hour after the beginning of the incubation, and intracellular staining was performed at 6 hours with mAbs specific for CD154 after surface CD3 and CD4 staining. To assess cytokine secretion and DC maturation, supernatants were harvested at 24 hours and cytokine concentration was measured by CBA. Cells were harvested at 48 hours using Trypsin-EDTA (Gibco) and stained with mAbs specific for CD86. Cells were analyzed by flow cytometry and CD86 MFI was assessed on gated DCs based on FSC and SSC criteria.

To assess the effect of CD4 T cell PD-1 blockade on CD8^+^ T cells, CD8^+^ cells were magnetically sorted from PBMCs from patients with OC, who we previously identified as responders to NY-ESO-1 with detectable CD8 T cell and antibody responses (7). Isolated CD8^+^ T cells (0.5 × 10^6^ to 1 × 10^6^ cells, per well) were cocultured with autologous iDCs (200,000 to 400,000 cells, per well), obtained from CD14^+^ cells as detailed above, and autologous CD4^+^ TILs (200,000 cells, per well) were pretreated or not with anti–PD-1 mAbs (10 g/mL), as described above, and rhIL2 (20 IU/mL) and stimulated with NY-ESO-1 peptides. Short NY-ESO-1 peptides (1 μM for HLA-A0201-restricted peptide and 0.5 μM for HLA-Cw0304–restricted and HLA-B3501–restricted peptides; Peptide 2.0 Inc.; Supplemental Table 5), selected according to patient HLA, were used to stimulate specific CD8 T cells and the NY-ESO-1 long peptide pool (1 μM) was used to stimulate specific CD4 Tconv TILs. Day 10 cultures were stained with HLA class I dextramers containing NY-ESO-1 peptides (Immudex) for 30 minutes at room temperature prior to anti-CD8 mAbs staining and analyzed by flow cytometry. HLA class I/peptide multimers are listed in Supplemental Table 6.

### Statistics.

Normality was assessed using the Shapiro-Wilk test. For normally distributed values, the 2-tailed *t* test was used in case of paired or unpaired data. When the values were not normally distributed, the comparison of variables was performed with Wilcoxon or Mann-Whitney *U* test for paired and unpaired data, respectively. For each test, a *P* value of less than 0.05 was considered significant. Analyses were performed with GraphPad Prism 7 software.

### Study approval.

Peripheral blood and tumor samples were collected from patients with head and neck, ovarian, and cervical cancer at the time of surgery at the Institut Universitaire du Cancer de Toulouse – Oncopole in accordance to the Declaration of Helsinki, upon approval by the Institutional Review Board (DC-2016-2656 and DECIdE protocol, NCT03958240) and signed informed consent.

## Author contributions

CCB designed research, conducted experiments, acquired and analyzed data, and wrote the manuscript. AS, CMS, and MM designed research, conducted experiments, acquired and analyzed the data, and participated in drafting the manuscript. CMG, CGR, VS, GF, LG, SV, ADB, EM, PR, JPD, and AM provided patient samples and analyzed clinical data. MT, FP, and JJF developed software and analyzed scRNA-Seq data. VS, CMG, and CV conducted experiments and acquired and analyzed data. CD and AM supervised research. MA designed and supervised research, analyzed the data, and wrote the manuscript. All authors reviewed the manuscript.

## Supplementary Material

Supplemental data

## Version 1 12/17/2020

In-Press Preview

## Version 2 01/25/2021

Electronic publication
