# Supplementary material for: PD-1 blockade restores helper activity of tumor-infiltrating, exhausted PD-1hiCD39+ CD4 T cells
Source: JCI Insight. 2021 Jan 25;6(2):e142513. doi: 10.1172/jci.insight.142513 (PMC7934837; doi:10.1172/jci.insight.142513)
Supplement: Supplemental data [file jciinsight-6-142513-s210.pdf]

Supplemental Data Materials

Supplemental Tables 1-6

Supplemental Figures 1-11

Supplemental Tables

Supplemental Table 1: List of flow cytometry antibodies

Antibody	Clone	Catalog number	Manufacturer	RRID
**CD3**	UCH-T1	555333	BD	AB_395740
**CD3**	UCH-T1	555332	BD	AB_395739
**CD3**	UCH-T1	563546	BD	AB_2744387
**CD3**	UCH-T1	563109	BD	AB_2732053
**CD3**	UCH-T1	555335	BD	AB_398591
**CD4**	RPA-T4	560768	BD	AB_1937323
**CD4**	RPA-T4	564724	BD	AB_2738917
**CD8**	RPA-T8	560774	BD	AB_1937325
**CD39**	TU66	563681	BD	AB_2738370
**CD45RA**	HI100	11-0458-42	eBioscience	AB_11219672
**CD49a**	SR84	559596	BD	AB_397288
**CD69**	FN50	560737	BD	AB_1727508
**CD86**	2331 (FUN1)	555658	BD	AB_396013
**CD103**	BER-ACT8	550259	BD	AB_393563
**CD152 (CTLA-4)**	BNI3	563931	BD	AB_2738491
**CD154**	TRAP1	555700	BD	AB_396050
**PD-1**	EH12.1	565299	BD	AB_2739167
**TIGIT**	MBSA43	25-9500-42	eBioscience	AB_2573548
**CD366 (TIM-3)**	7D3	565562	BD	AB_2744369
**TOX**	TXRX10	50-6502-82	eBioscience	AB_2574265
**IFN-γ**	B27	557995	BD	AB_396977
**TNF-α**	MAb1	557647	BD	AB_396764
**FOXP3**	PC5101	56-4776-41	eBioscience	AB_1582210
**FOXP3**	PCH101	11-4776-42	eBioscience	AB_1724125

Supplemental Table 2: List of antibodies for FACS cell sorting

Antibody	Clone	Catalog number	Manufacturer	RRID
**CD3**	UCH-T1	561027	BD	AB_10561682
**CD4**	RPA-T4	555346	BD	AB_395751
**CD8**	RPA-T8	560774	BD	AB_1937325
**CD39**	TU66	563681	BD	AB_2738370
**PD-1**	EH12.1	563789	BD	AB_2738425
**CD127**	HIL-7R-M21	560551	BD	AB_1645548
**CD25**	B1.49.9	B09684	Beckman Coulter	AB_2861133

Supplemental Table 3: List of NY-ESO-1 long peptides for CD4 T-cell stimulation

Peptide	Sequence
**NY-ESO-1$_{1\text{-}20}$**	MQAEGRGTGGSTGDADGPGG
**NY-ESO-1$_{11\text{-}30}$**	STGDADGPGGPGIPDGPGGN
**NY-ESO-1$_{21\text{-}40}$**	PGIPDGPGGNAGGPGEAGAT
**NY-ESO-1$_{31\text{-}50}$**	AGGPGEAGATGGRGPRGAGA
**NY-ESO-1$_{41\text{-}60}$**	GGRGPRGAGAARASGPGGGA
**NY-ESO-1$_{51\text{-}70}$**	ARASGPGGGAPRGPHGGAAS
**NY-ESO-1$_{61\text{-}80}$**	PRGPHGGAASGLNGCCRCGA
**NY-ESO-1$_{71\text{-}90}$**	GLNGCCRCGARGPESRLLEF
**NY-ESO-1$_{81\text{-}100}$**	RGPESRLLEFYLAMPFATPM
**NY-ESO-1$_{91\text{-}110}$**	YLAMPFATPMEAELARRSLA
**NY-ESO-1$_{101\text{-}120}$**	EAELARRSLAQDAPPLPVPG
**NY-ESO-1$_{111\text{-}130}$**	QDAPPLPVPGVLLKEFTVSG
**NY-ESO-1$_{119\text{-}143}$**	PGVLLKEFTVSGNILTIRLTAADHR
**NY-ESO-1$_{131\text{-}150}$**	NILTIRLTAADHRQLQLSIS
**NY-ESO-1$_{139\text{-}160}$**	AADHRQLQLSISSCLQQLSLLM
**NY-ESO-1$_{151\text{-}170}$**	SCLQQLSLLMWITQCFLPVF
**NY-ESO-1$_{161\text{-}180}$**	WITQCFLPVFLAQPPSGQRR

Supplemental Table 4: List of anti-HLA class II functional antibodies

Antibody	Clone	Catalog number	Manufacturer	RRID
**HLA-DP**	B7/21	H260	Leinco Technologies	AB_2737518
**HLA-DQ**	SPVL3	IM0416	Beckman Coulter	AB_2861134
**HLA-DR**	G46-6	555809	BD	AB_396143

Supplemental Table 5: List of NY-ESO-1 short peptides for CD8 T-cell stimulation

Peptide	Sequence
**NY-ESO-1$_{92\text{-}100}$**	LAMPFATPM
**NY-ESO-1$_{157\text{-}165A}$**	SLLMWITQA
**NY-ESO-1$_{94\text{-}102}$**	MPFATPMEA
**NY-ESO-1$_{94\text{-}104}$**	MPFATPMEAEL
**NY-ESO-1$_{96\text{-}104}$**	FATPMEAEL

Supplemental Table 6: List of HLA class I/peptide multimers

Peptide	HLA	Multimer type	Catalog number	Manufacturer
**NY-ESO-1$_{157\text{-}165A}$**	A*0201	Dextramer	WB-3247-PE	Immudex
**NY-ESO-1$_{94\text{-}102}$**	B*3501	Dextramer	WK2701-PE	Immudex
**NY-ESO-1$_{94\text{-}104}$**	B*3501	Dextramer	WK5341-PE	Immudex
**NY-ESO-1$_{92\text{-}100}$**	Cw*0304	Dextramer	WS3694-PE	Immudex
**NY-ESO-1$_{96\text{-}104}$**	Cw*0304	Dextramer	WS5342-PE	Immudex

Figures

Supplemental Figure 1

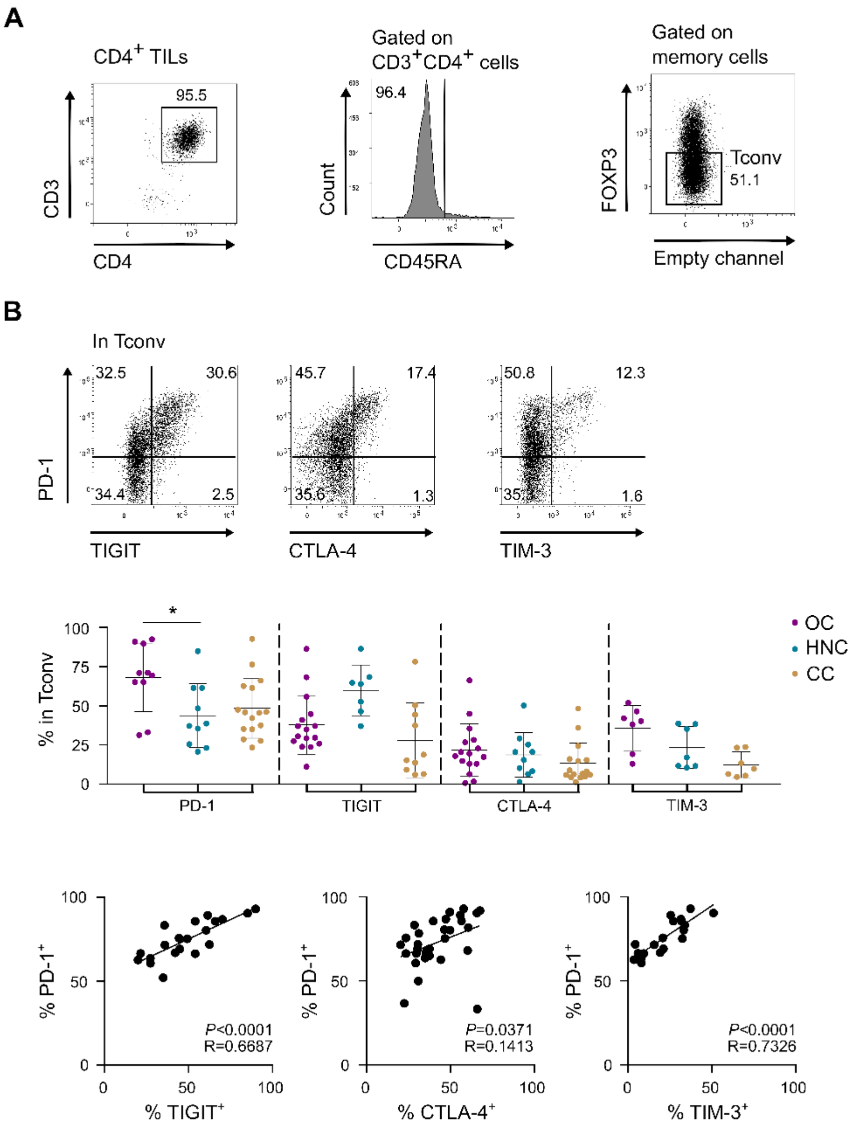

Supplemental Figure 1. Immune checkpoints expression in CD4 Tconv TILs. Isolated CD4+ TILs were stained ex vivo with fluorochrome-labeled mAbs specific for CD3, CD4, CD45RA, PD-1, TIGIT, CTLA-4, TIM-3 and FOXP3 and analyzed by flow cytometry. (**A**) Gating strategy for CD4 Tconv TILs assessment. Left dot plot shows CD3 versus CD4 expression in CD4+ TILs, center histogram plot shows CD45RA expression in gated CD3+CD4+ T cells and right dot plot shows FOXP3 expression in gated memory (CD45RA−) CD4+ T cells where CD4 Tconv are gated as FOXP3− cells. (**B**) Dot plots show PD-1 versus TIGIT, CTLA-4 and TIM-3 expression in gated memory CD4 Tconv TILs. Proportions of PD-1+, TIGIT+, CTLA-4+ and TIM-3+ cells in CD4 Tconv are summarized for ovarian (OC, *n=10,* purple), head and neck (HNC, *n=16,* green) and cervical (CC, *n=7,* yellow) cancer patients (middle). Correlation between the proportions of PD-1+ and those of TIGIT+ (*n=19*), CTLA-4+ (*n=31*) or TIM-3+ (*n=19*) CD4 Tconv TILs from the three tumor types (bottom). Data are presented as mean ± SD. Mann-Whitney test (middle) and Pearson correlation (bottom) were used to compare variables (**B**).

Supplemental Figure 2

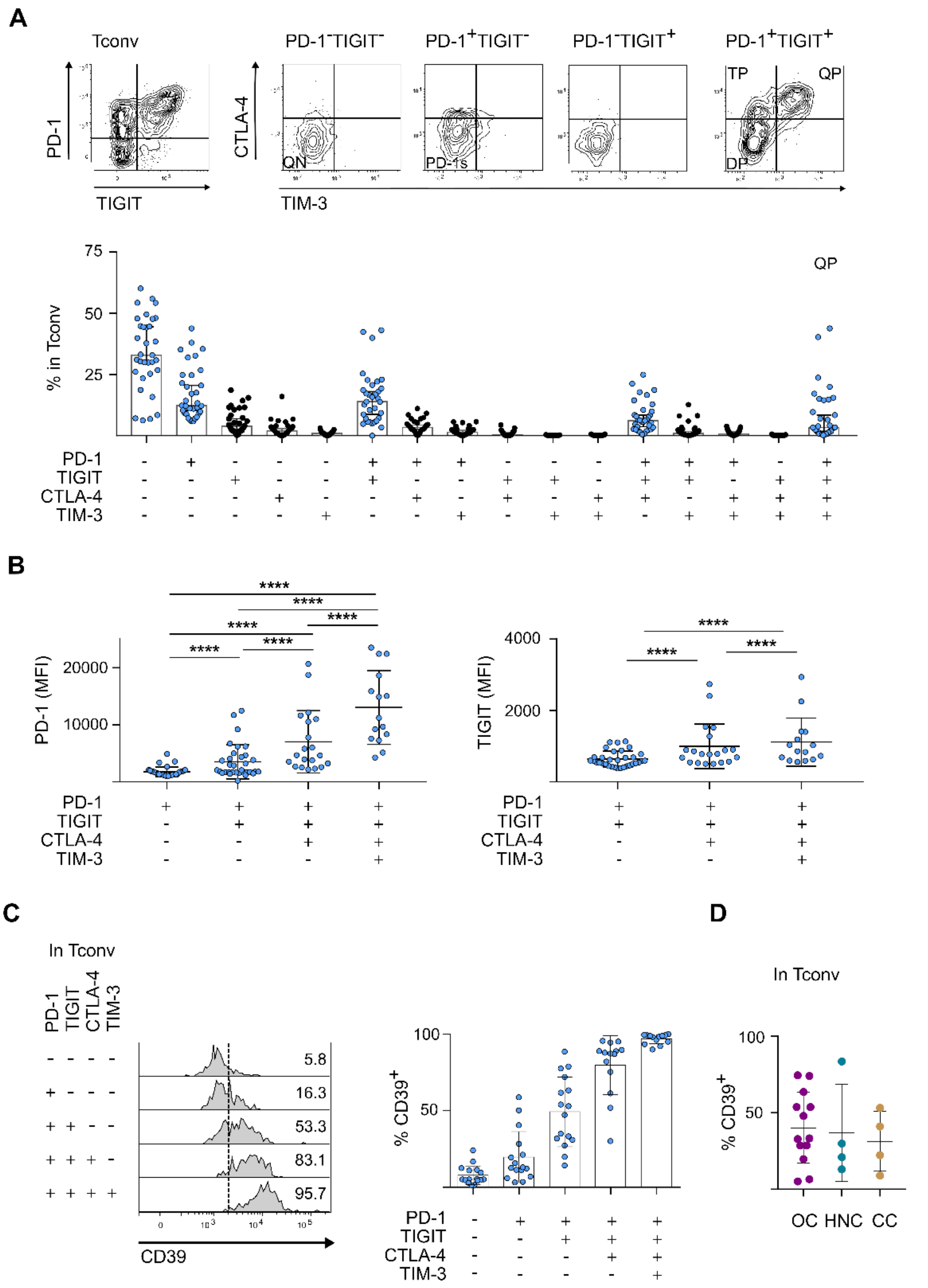

Supplemental Figure 2. Co-expression of immune checkpoints and CD39 defines distinct CD4 Tconv TIL subsets. Isolated $CD4^+$ TILs were stained ex vivo with mAbs specific for CD3, CD4, CD45RA, PD-1, TIGIT, CTLA-4, TIM-3, CD39 and FOXP3 and analyzed by flow cytometry. (**A**) Contour plots (right), showing CTLA-4 and TIM-3 expression, are gated on $PD\text{-}1^-TIGIT^-$, $PD\text{-}1^+TIGIT^-$, $PD\text{-}1^-TIGIT^+$ and $PD\text{-}1^+TIGIT^+$ CD4 Tconv populations as shown in the left contour plot. Proportions of each of the 16 possible combinations of immune checkpoint (IC) expression are summarized (*n=33*). Tconv TIL subpopulations with a median proportion > 5% are represented in blue. QP, quadruple positive cells expressing the 4 ICs, i.e. $PD\text{-}1^+TIGIT^+CTLA\text{-}4^+TIM\text{-}3^+$ Tconv TILs. (**B**) Mean fluorescence intensity (MFI) of PD-1 and TIGIT in CD4 Tconv TIL subpopulations defined in A (with a median proportion > 5%) expressing PD-1 or TIGIT, respectively (numbers of samples are as in **A**). (**C**) Histogram plots showing CD39 expression in CD4 Tconv TIL subpopulations identified in **A** as representing > 5% of the total population. Proportions of $CD39^+$ cells in each subpopulation are summarized (*n=16*). (**D**) Proportions of $CD39^+$ cells in CD4 Tconv are summarized for ovarian (OC, *n=13,* purple), head and neck (HNC, *n=4,* green) and cervical (CC, *n=4,* yellow) cancer patients. Data are presented as mean ± SD. Wilcoxon test or two-tailed paired test were used to compare variables (**B**). **, $P < 0.01$; ***, $P < 0.001$; ****, $P < 0.0001$.

Supplemental Figure 3

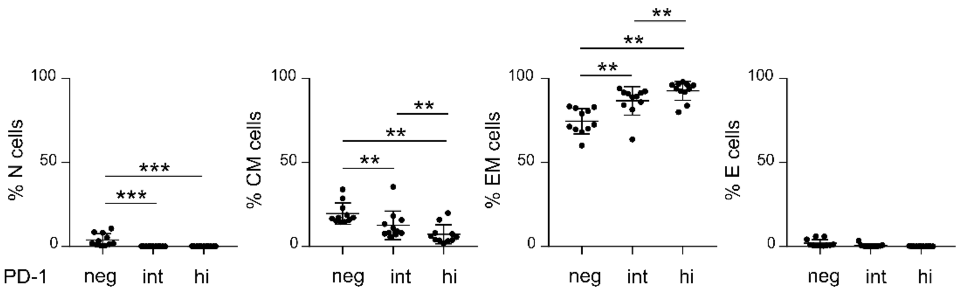

Supplemental Figure 3. PD-1^{hi} CD4 Tconv TILs are effector memory T cells. $CD4^+$ TILs were stained ex vivo with mAbs specific for CD3, CD4, CD45RA, CCR7 and PD-1 and the percentages of naïve (N, $CD45RA^+CCR7^+$), central memory (CM, $CD45RA^-CCR7^+$), effector memory (EM, $CD45RA^-CCR7^-$), and effector (E, $CD45RA^+CCR7^-$) cells among PD-1^-, PD1^{int} and PD1^{hi} subsets are represented (*n=11*). Data are presented as mean ± SD. Wilcoxon test was used to compare variables. **, $P < 0.01$; ***, $P < 0.001$; ****, $P < 0.0001$.

Supplemental Figure 4

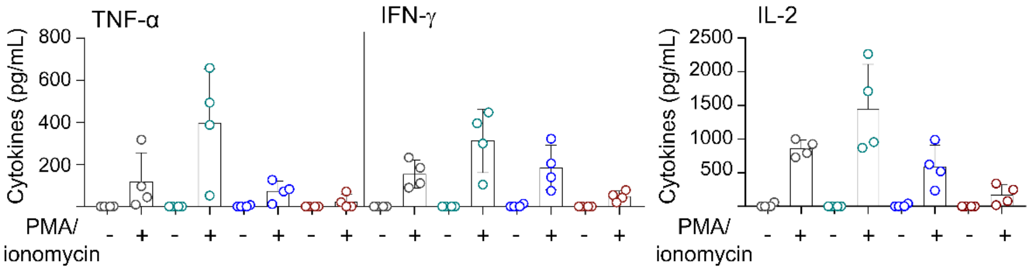

Supplemental Figure 4. PD-1^hi^CD39^+^ CD4 Tconv TILs have reduced cytokine production capacities. CD4$^+$ TILs were stained ex vivo with mAbs specific for CD3, CD4, CD8, CD127, CD25, PD-1 and CD39, and CD4 Tconv (CD3$^+$CD8$^-$CD4$^+$CD25$^-$CD127$^+$) were sorted into PD-1$^-$CD39$^-$(grey), PD-1^{lo}CD39$^-$(green), PD-1^{hi}CD39$^-$(blue) and PD-1^{hi}CD39$^+$ (red) subsets that were stimulated or not in vitro with PMA/ionomycin overnight. TNF-α, IFN-γ and IL-2 were quantified by ELISA in the supernatant (*n=4*). Data are presented as mean ± SD.

Supplemental Figure 5

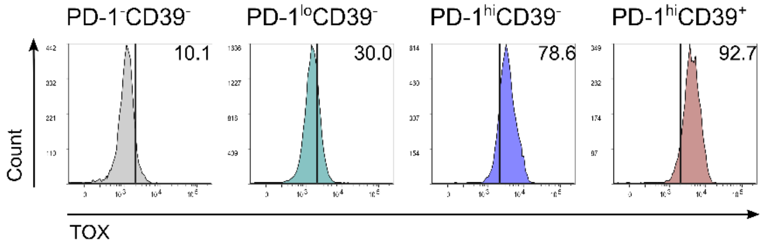

Supplemental Figure 5. TOX expression in CD4 Tconv TIL subsets. Isolated CD4$^+$ TILs were stained ex vivo with mAbs specific for CD3, CD4, CD45RA, PD-1, CD39, TOX and FOXP3 and analyzed by flow cytometry. Examples of histogram plots showing TOX expression in CD4 Tconv TIL populations defined according to PD-1 and CD39 expression; PD-1$^-$CD39$^-$, PD-1^{lo}CD39$^-$, PD-1^{hi}CD39$^-$ and PD-1^{hi}CD39$^+$.

Supplemental Figure 6

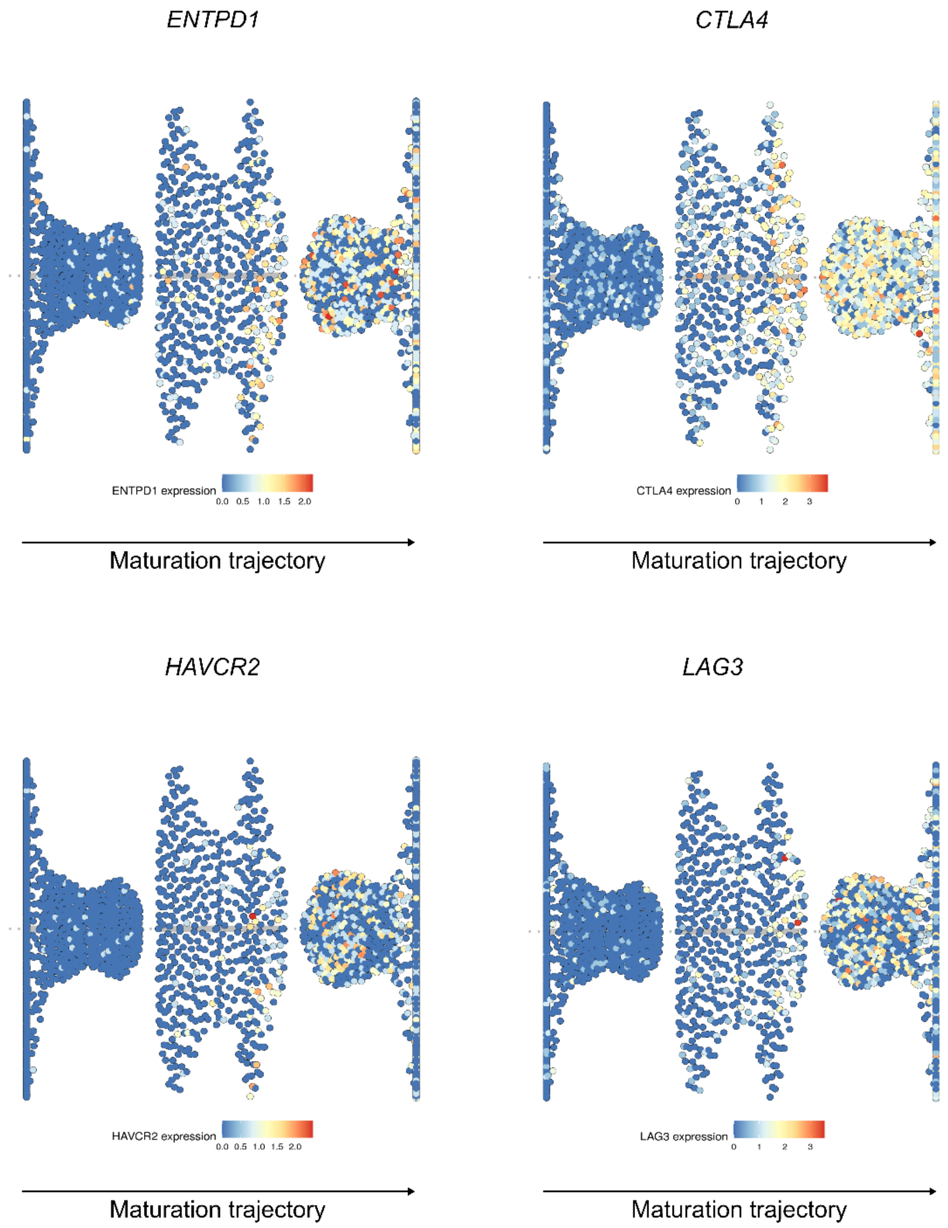

Supplemental Figure 6. Pseudotime analysis of scRNA-seq data. Pseudotime maturation trajectory of CD4 Tconv based on scRNA-Seq data presented in dendrograms color-coded according to *ENTPD1*, *CTLA4*, *HAVCR2* and *LAG3* gene expression levels (*n=4*).

Supplemental Figure 7

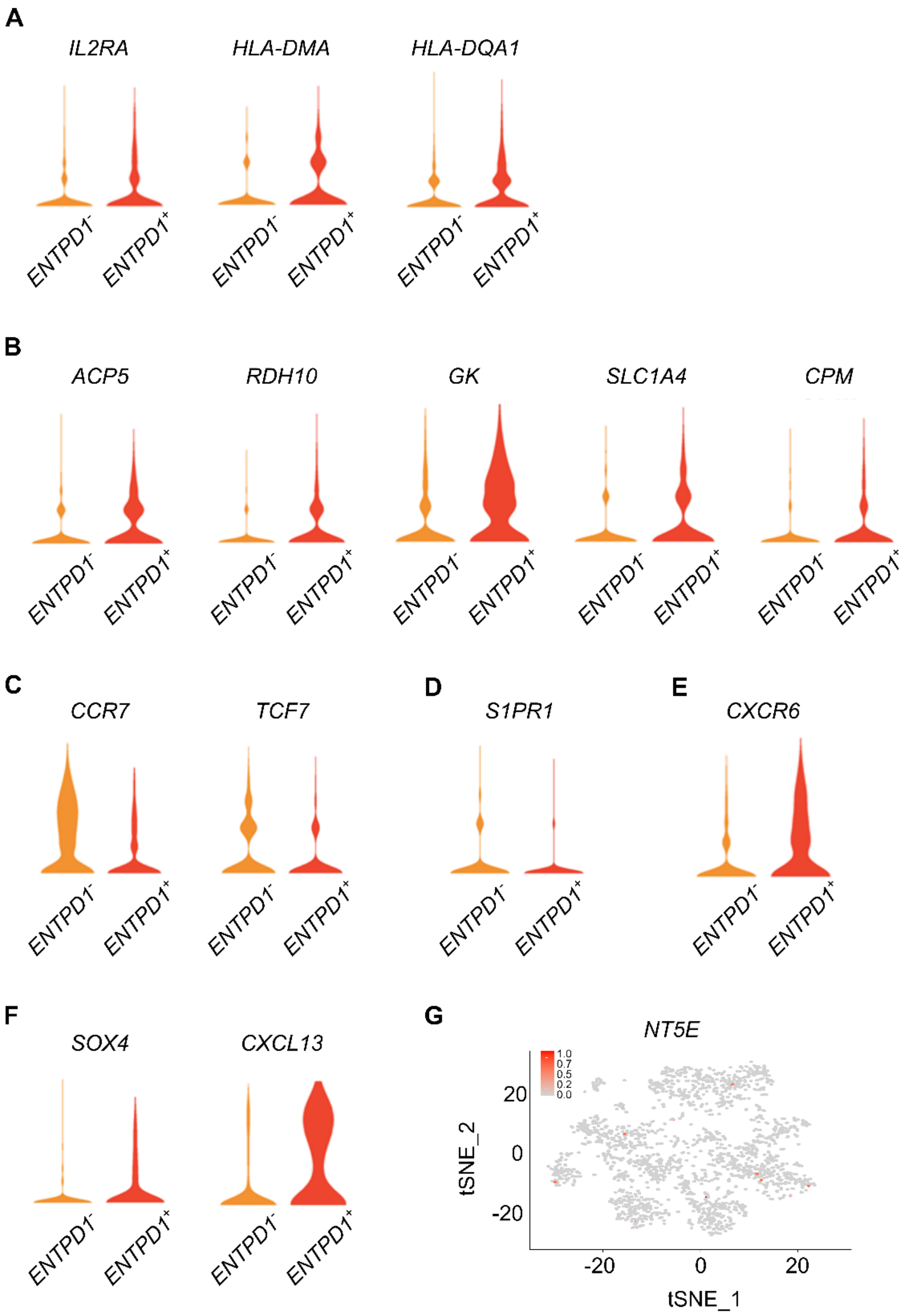

Supplemental Figure 7. *ENTPD1*⁺ CD4 Tconv TILs exhibit features of T-cell activation, late differentiation and tumor residency. Differential gene expression analysis between *ENTPD1*⁺ and *ENTPD1*⁻ CD4 Tconv was performed using scRNA-Seq data (Figure 3B). (**A-F**) Violin plots of expression of 14 genes shown to be significantly differentially expressed in *ENTPD1*⁺ versus *ENTPD1*⁻ CD4 Tconv TILs (*n=4*). (**G**) t-SNE plot color-coded by levels of expression (grey to red) of *NT5E* (CD73) gene.

Supplemental Figure 8

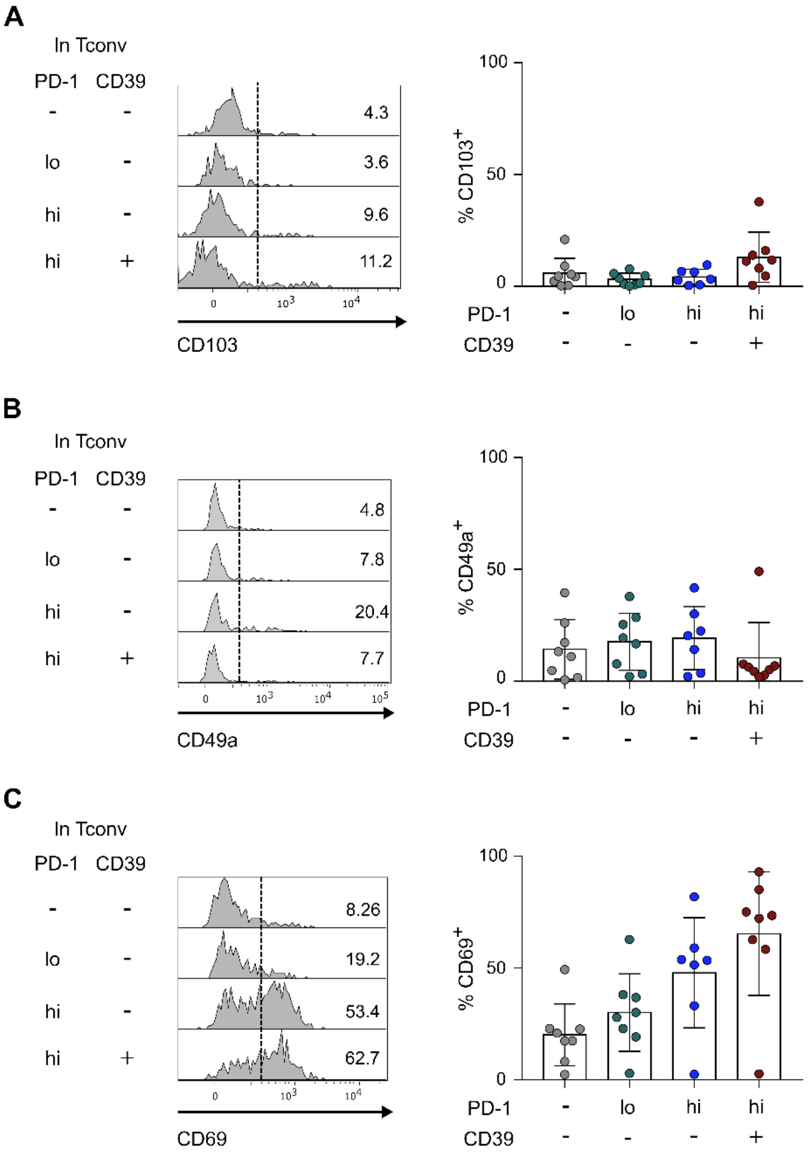

Supplemental Figure 8. Trm markers expression in CD4 Tconv TIL subsets. Isolated $CD4^+$ TILs were stained ex vivo with mAbs specific for CD3, CD4, CD45RA, PD-1, CD39, CD49a, CD69, CD103 and FOXP3 and analyzed by flow cytometry. (**A-C**) Examples of histogram plots showing CD103 (**A**), CD49a (**B**) and CD69 (**C**) expression in CD4 Tconv TIL populations defined according to PD-1 and CD39 expression; PD-1^-CD39^-, PD-1^{lo}CD39^-, PD-1^{hi}CD39^- and PD-1^{hi}CD39^+ (*n=8*). Data are presented as mean ± SD.

Supplemental Figure 9

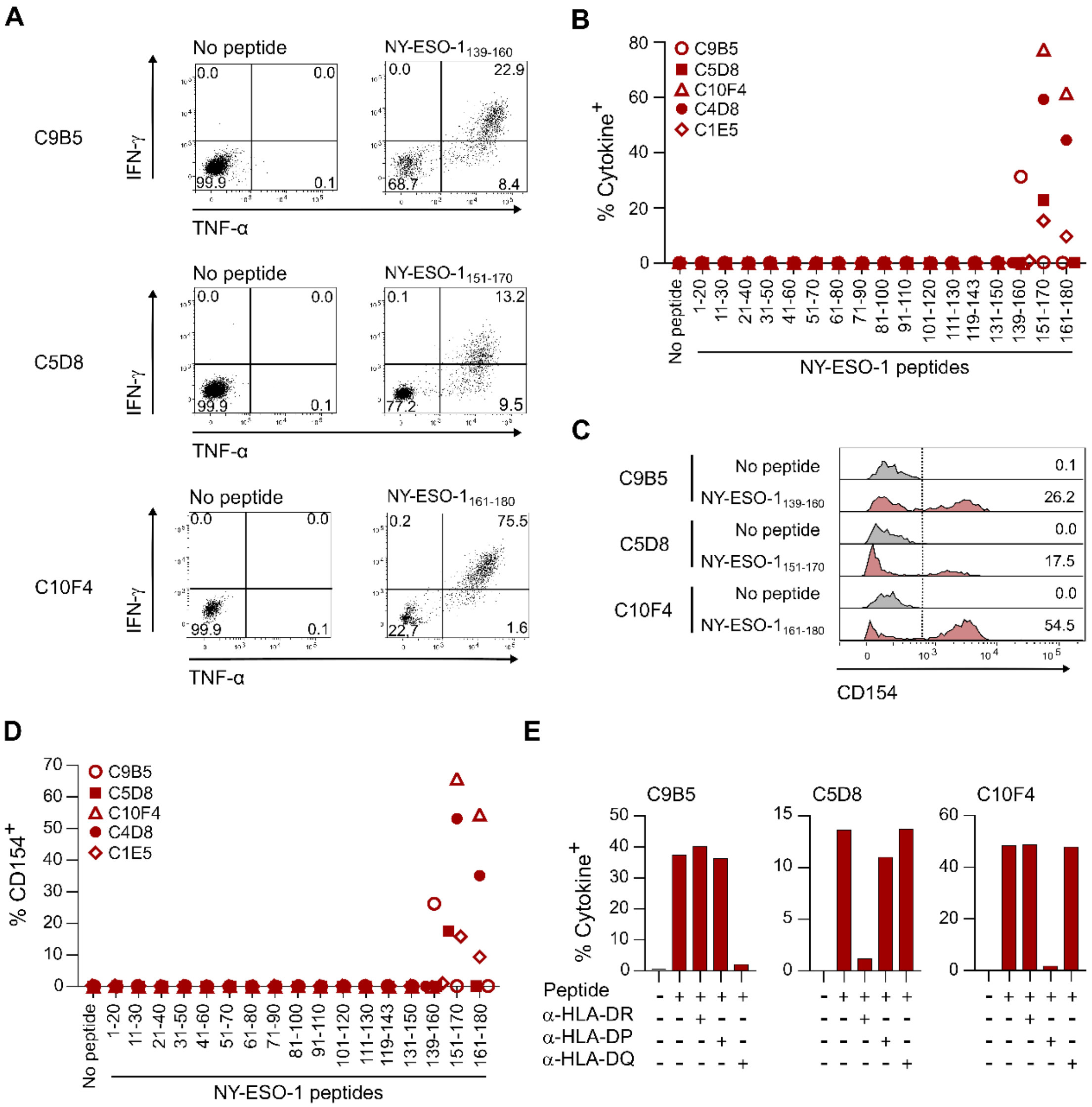

Supplemental Figure 9. Fine specificity and MHC restriction of PD-1^{hi}CD39^{+} Tconv TILs-derived NY-ESO-1-specific clones. (**A**-**D**) PD-1^{hi}CD39^{+} CD4 Tconv TILs-derived clones responding to NY-ESO-1 peptide pool (Figure 4C) were stimulated or not with each single peptide for 4 hours in the presence of HLA-matched iDCs from HDs and IFN-γ, TNF-α and CD154 expression were analyzed by intracellular staining and flow cytometry. (**A**) Examples of dot plots showing IFN-γ versus TNF-α production of the indicated NY-ESO-1–specific clones in the absence or presence of the indicated NY-ESO-1 peptides (**B**) Proportions of cytokine^{+} (IFN-γ and or TNF-α) cells after stimulation or not of the five NY-ESO-1–specific clones with single NY-ESO-1 peptides (**C**) Examples of histogram plots showing CD154 expression in the indicated NY-ESO-1–specific clones in the absence or presence of the indicated NY-ESO-1 peptides. (**D**) Proportions of CD154^{+} cells after stimulation or not of the five NY-ESO-1–specific clones with single NY-ESO-1 peptides. (**E**) Proportions of cytokine^{+} (IFN-γ and/or TNF-α) cells after stimulation with HLA-matched iDCs from HDs with corresponding NY-ESO-1 single peptides for C9B5, C5D8 and C10F4 clones, in the presence or absence of blocking anti-HLA-DR, anti-HLA-DP and anti-HLA-DQ mAbs. (**B**,**D**,**E**) One experiment representative of two independent experiments.

Supplemental Figure 10

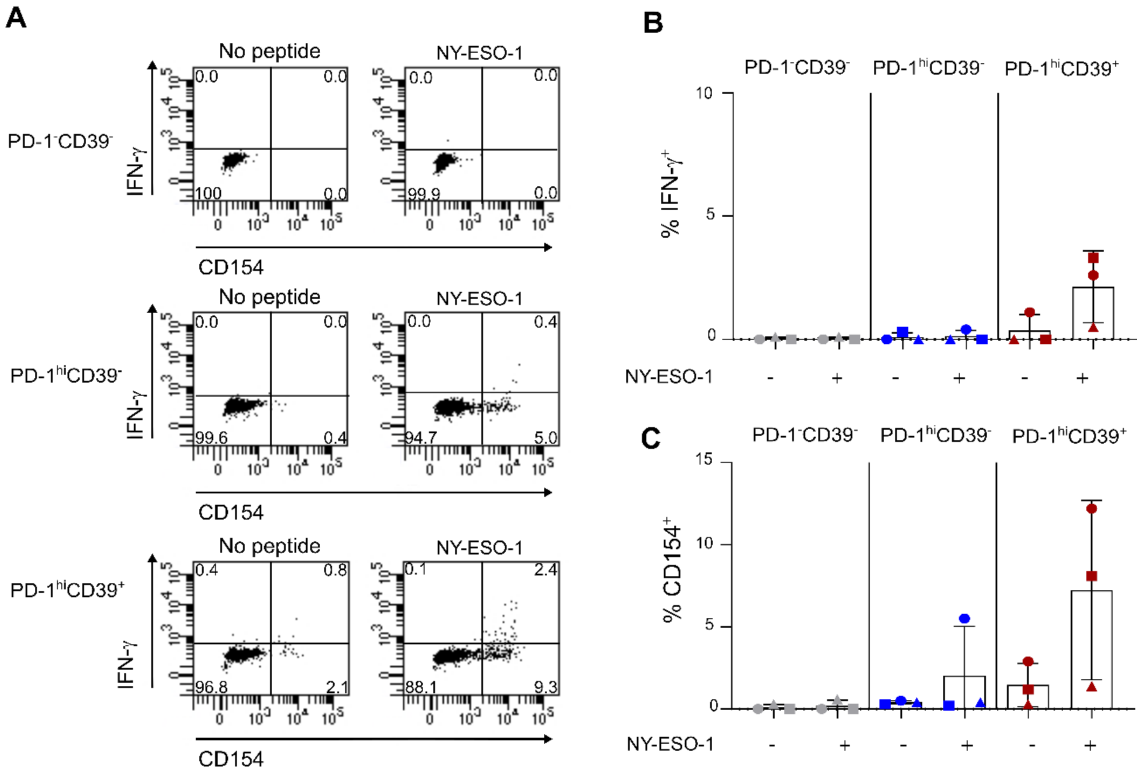

Supplemental Figure 10. Ag specificity of Tconv TIL subets. Ex vivo isolated CD4 TILs (*n=3*) were stimulated or not for 6 hours with the NY-ESO-1 peptide pool in presence of autologous circulating $CD14^+$ cells and IFN-γ and CD154 expression were analyzed by intracellular staining and flow cytometry. (**A**) Dot plots show IFN-γ versus CD154 expression for the $PD\text{-}1^{-}CD39^{-}$, $PD\text{-}1^{hi}CD39^{-}$ and $PD\text{-}1^{hi}CD39^{+}$ Tconv subsets ($CD3^+CD4^+FOXP3^-$) in presence or absence of the NY-ESO-1 peptide pool. (**B, C**) Proportions of IFN-γ^+ and $CD154^+$ cells in the three subsets are summarized. Data are presented as mean ± SD.

Supplemental Figure 11

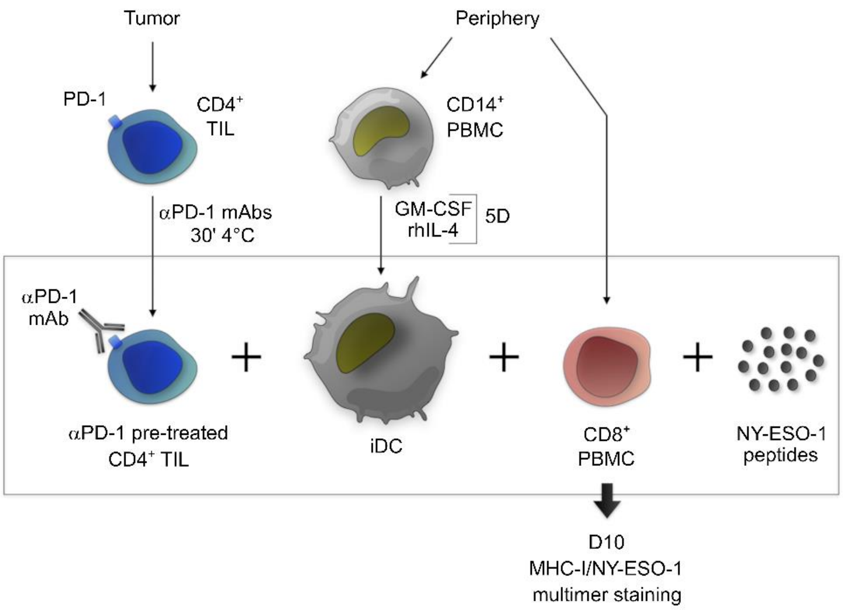

Supplemental Figure 11. Graphical representation of circulating CD8 T-cell stimulation experiment with NY-ESO-1 peptides in the presence of autologous CD4$^+$ TILs and iDCs. CD14$^+$ cells were sorted from PBMCs of ovarian cancer patients exhibiting antibody and CD8 T-cell responses to the NY-ESO-1 Ag and were differentiated into iDCs by culture for 5 days in the presence of GM-CSF and rhIL-4. At day 5, CD4$^+$ TILs were isolated from the same patients, pre-incubated or not with anti-PD-1 mAbs for 30 minutes at 4°C and washed twice. In parallel, autologous circulating CD8 T cells were sorted from PBMCs and co-cultured with iDCs, anti-PD-1 pre-treated or non-pre-treated CD4$^+$ TILs in the presence of NY-ESO-1 peptides, as detailed in Materials and Methods, and rhIL-2. At day 10, cells were stained with MHC-I/NY-ESO-1 multimers and analyzed by flow cytometry.
